# *CrystalExplorer* model energies and energy frameworks: extension to metal coordination compounds, organic salts, solvates and open-shell systems

**DOI:** 10.1107/S205225251700848X

**Published:** 2017-07-04

**Authors:** Campbell F. Mackenzie, Peter R. Spackman, Dylan Jayatilaka, Mark A. Spackman

**Affiliations:** a School of Molecular Sciences, University of Western Australia, Perth, 6009, Australia

**Keywords:** *CrystalExplorer*, model energies, energy frameworks, coordination compounds, open-shell systems, intermolecular interactions

## Abstract

The application domain of accurate and efficient CE-B3LYP and CE-HF model energies for intermolecular interactions in molecular crystals is extended by calibration against density functional results for 1794 molecule/ion pairs extracted from 171 crystal structures. The mean absolute deviation of CE-B3LYP model energies from DFT values is a modest 2.4 kJ mol^−1^ for pairwise energies that span a range of 3.75 MJ mol^−1^. The new sets of scale factors determined by fitting to counterpoise-corrected DFT calculations result in minimal changes from previous energy values. Coupled with the use of separate polarizabilities for interactions involving monatomic ions, these model energies can now be applied with confidence to a vast number of molecular crystals. Energy frameworks have been enhanced to represent the destabilizing interactions that are important for molecules with large dipole moments and organic salts. Applications to a variety of molecular crystals are presented in detail to highlight the utility and promise of these tools.

## Abstract 2

The accurate and efficient CE-B3LYP and CE-HF model energies for intermolecular interactions in molecular crystals are extended to a broad range of crystals by calibration against density functional results for molecule/ion pairs extracted from 171 crystal structures. The mean absolute deviation of CE-B3LYP model energies from DFT values is a modest 2.4 kJ mol^−1^ for pairwise energies that span a range of 3.75 MJ mol^−1^.

## 1. Introduction   

The detailed analysis of the interactions between molecules and ions in crystals plays an increasingly important role in modern solid-state chemistry and, in particular, crystal engineering, where the derivation of predictive structure–property relationships is key to a genuine ‘engineering’ of crystals. This, of course, was articulated some time ago by Desiraju (1989 ▸), who described crystal engineering as the ‘understanding of intermolecular interactions in the context of crystal packing and the utilization of such understanding in the design of new solids with desired physical and chemical properties’. Utilization and design require understanding as an essential precursor, and the context of crystal packing in this statement is especially important. Recent years have witnessed a rapid growth of publications that focus on the relative importance of *noncanonical* interactions (*e.g.* halogen, chalcogen, pnicogen and tetrel bonds), but it is not obvious that they have enhanced our understanding of the relationship between the structure of molecules (geometric and electronic), the crystal structures they form and their consequent chemical and physical properties. It seems pertinent to ask whether we are converging on the requisite intimate, and ultimately useful, understanding of why molecules and ions are arranged in crystals as observed, or merely cataloguing an increasing number of examples of relatively weak intermolecular ‘interactions’ while ignoring their common origins?

The recent series of essays published in this journal (Dunitz, 2015 ▸; Lecomte *et al.*, 2015 ▸; Thakur *et al.*, 2015 ▸) has highlighted some of the issues confronting the field at present, as well as exemplifying two different ways of looking at intermolecular interactions: one with an emphasis on specific atom–atom contacts (or interactions, or bonds), the other a whole-of-molecule approach that is blind to atom–atom interactions. A large number of computational approaches have been exploited in recent crystal structure analyses in efforts to investigate and understand the nature, strength and importance of various intermolecular interactions in crystals, and it is instructive to summarize a small number of these. For example, Parsons and colleagues identified ‘the haza­rds of over-simplifying intermolecular interactions on the basis of prominent atom–atom contacts’ as part of an analysis of the high-pressure polymorph ∊-glycine (Moggach *et al.*, 2015 ▸). That analysis made use of Gavezzotti’s PIXEL methodology (Gavezzotti, 2005 ▸) and symmetry-adapted perturbation theory to compute intermolecular energies, Hirshfeld surface analysis (Spackman & Jayatilaka, 2009 ▸) and periodic DFT calculations, as well as mapping the molecular electrostatic potential (ESP) on molecular Hirshfeld surfaces to illustrate the electrostatic complementarity (or otherwise) between neighouring molecules. Hirshfeld surface analysis, ESP mapping and Quantum Theory of Atoms in Molecules (QTAIM) (Bader, 1990 ▸) topological analysis of theoretical and experimental electron densities were employed by Pyziak *et al.* (2015 ▸) as part of their experimental charge–density investigation of intermolecular interactions, including chalcogen bonding, in 4-[[4-(methoxy)-3-quinolinyl]thio]-3-thiomethylquinoline, and by Lai *et al.* (2016 ▸) in a study comparing β-piroxicam with piroxicam monohydrate. The latter work also reported intermolecular interaction energies estimated from the experimental potential energy densities at bond-critical points (Espinosa *et al.*, 1998 ▸), an approach that reflects the atom–atom perspective on intermolecular interactions. Although the resulting energy estimates from this approach have been shown to be unreliable, and for a very wide range of interactions (Spackman, 2015 ▸), they are commonly reported in current studies, for example, by Zhurov & Pinkerton (2015 ▸) as part of their experimental charge–density analysis of 2-nitro­benzoic acid, and by Landeros-Rivera *et al.* (2016 ▸), who examined intermolecular interactions in crystalline arene–perhaloarene adducts by QTAIM methods, ESP mapping and noncovalent index (NCI) surfaces (Johnson *et al.*, 2010 ▸), as well as energies from Gavezzotti’s PIXEL method for selected molecular pairs. As a final example, we mention the comprehensive study of intermolecular interactions in six polymorphs of phenobarbital by Gelbrich *et al.* (2016 ▸), who analysed PIXEL-derived energies and lattice energy contributions in great detail.

Two decades ago, Desiraju argued for a ‘compelling need…to be able to visualize a crystal structure in its entirety, not just look at selected intermolecular interactions which have been deemed to be important’ (Desiraju, 1997 ▸) and a year later Nangia & Desiraju (1998 ▸) noted that ‘a detailed understanding of crystal packing and crystal design depends very substantially on viewing the molecule as an organic whole’. We wholeheartedly embrace these sentiments and the focus of our research since then, especially the publications and the embodiment of all original ideas and tools in *CrystalExplorer*, constitutes an attempt to venture beyond the outdated paradigm identified by Desiraju, and to view molecules as ‘organic wholes’, thereby fundamentally altering the discussion of intermolecular interactions through the use of a variety of novel computational and graphical tools (Spackman, 2013 ▸; Turner *et al.*, 2011 ▸; Spackman & Jayatilaka, 2009 ▸).

As part of our ongoing research, we recently described a computationally inexpensive approach to obtaining accurate intermolecular interaction energies for organic (and some inorganic) molecular crystals (Turner *et al.*, 2014 ▸), and their use in constructing ‘energy frameworks’ that offer a powerful new way to visualize the supramolecular architecture of molecular crystal structures (Turner *et al.*, 2015 ▸). Applications of these new tools have so far been limited (Eikeland *et al.*, 2016*a* ▸,*b* ▸; Dey *et al.*, 2016*a* ▸,*b* ▸; Shi *et al.*, 2015 ▸), in part because of the limited release of the software to date, and also because the original development focused solely on the calibration of the model energies against DFT results for crystals comprising neutral organic and *p*-block inorganic molecules. This limitation has provided the motivation for the present work, which outlines how the approach can now be applied with confidence to molecular crystals comprising metal coordination com­pounds, organic salts, solvates and open-shell molecules.

As indicated in the original presentation of these model energies, our approach is inspired by Gavezzotti’s PIXEL method (Maschio *et al.*, 2011 ▸; Gavezzotti, 2002 ▸, 2003 ▸, 2005 ▸, 2008 ▸), which is increasingly used when applied to organic molecular crystals. Its extension to transition-metal coordination compounds was recently reported by Maloney *et al.* (2015 ▸). Our expression for the interaction energy between pairs of molecules is essentially the same as that used by Gavezzotti and many others, *viz.*

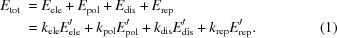
The breakdown of the interaction energy in this manner has been used extensively in energy-decomposition methods *via* variational [*e.g.* Kitaura & Morokuma (1976 ▸) and Ziegler & Rauk (1979 ▸)] and perturbation-based approaches [*e.g.* Hayes & Stone (1984 ▸) and Jeziorski *et al.* (1994 ▸)]. In the present work, 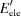, the classical electrostatic energy of interaction between monomer charge distributions, and 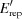, the exchange–repulsion energy, are obtained from the antisymmetric product of the monomer spin orbitals as described by Su & Li (2009 ▸). The polarization energy, 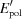, is estimated as a sum over atoms with terms of the kind −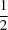
α|***F***|^2^, where the electric field ***F*** is computed at each atomic nucleus from the charge distribution of the other monomer and α are isotropic atomic polarizabilities (Thakkar & Lupinetti, 2006 ▸). The dispersion energy term, 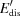, is Grimme’s D2 dispersion correction (Grimme, 2006 ▸) summed over all intermolecular atom pairs. As described below, the scale factors *k*_ele_, *etc*., in equation (1) are determined by calibration against quantum mechanical results.

Although our model energy formalism is similar in many ways to Gavezzotti’s PIXEL approach, it is important to recognize the significant differences. Individual energy terms in that approach depend on a fine-grained discrete representation of the molecular electron density as a sum of charged pixel volumes (actually voxels). The Coulombic energy between unperturbed molecular charge densities is fundamentally the same in the two methods (if based on the same wavefunctions), but obviously computed numerically in PIXEL. All other terms in the PIXEL approach make use of the same voxel breakdown and incorporate a set of atomic polarizabilities, as well as adjustable parameters, to account for short separations, damping of dispersion energies, and a scale factor and power-law dependence for the repulsion energy. These parameters are optimized to minimize the deviation between computed lattice energies and experimental sublimation enthalpies for a representative set of organic crystal structures. In contrast to PIXEL, our polarization energy incorporates accurate electric fields computed from monomer wavefunctions (but depends very much on the chosen isotropic atomic polarizabilities), our dispersion term is similar to that used increasingly in many ‘DFT+dispersion’ quantum chemical formalisms, and our repulsion term is computed from the quantum mechanical overlap of spin orbitals, rather than electron distributions. Like PIXEL, there are adjustable parameters to optimize, but rather than use experimental sublimation enthalpies, we determine the four scale factors in equation (1) by fitting to a large set of pairwise interaction energies obtained from theory, namely counterpoise-corrected B3LYP-D2/6-31G(d,p) energies obtained at the identical geometry. Both calibration approaches have limitations of course. Experimental sublimation enthalpies are known for several thousand organic and organometallic molecular crystals (Acree & Chickos, 2016 ▸, 2017 ▸), with estimated uncertainties typically ∼5 kJ mol^−1^ for organics and ∼24 kJ mol^−1^ for organometallics (Chickos, 2003 ▸). Experimental sublimation enthalpies are temperature dependent and smaller in magnitude than the corresponding lattice energy by approximately 2*RT* (Gavezzotti & Filippini, 1997 ▸; Maschio *et al.*, 2011 ▸; Otero-de-la-Roza & Johnson, 2012 ▸). Computed lattice energies can also depend significantly on the temperature of the crystal structure determination, and their comparison with experimental data often implicitly assumes no geometry change between the crystal and gas phase. On the other hand, the present counterpoise-corrected B3LYP-D2 energies use a relatively limited basis set, which results in quite a large basis set superposition error (BSSE) for some close geometries, but we have also encountered limitations of B3LYP itself, as compared to a more rigorous correlation method like MP2. In the following section, we discuss examples of these limitations, along with ways in which we have resolved or minimized their influence on the final results.

Given this recognition of inherent uncertainties it is clear that their use in investigating polymorphic systems is unlikely to be fruitful. In their extensive computational study of a large number of polymorph pairs, Nyman & Day (2015 ▸) concluded: ‘polymorphic lattice energy differences are typically very small: over half of polymorph pairs are separated by less than 2 kJ mol^−1^ and lattice energy differences exceed 7.2 kJ mol^−1^ in only 5% of cases. Unsurprisingly, vibrational contributions to polymorph free energy differences at ambient conditions are dominated by entropy differences’. This sort of accuracy is clearly unachieveable with PIXEL or CE-B3LYP model energies, and we must not expect it.

## 2. Methods   

### 2.1. Choice of crystal structures   

The training set chosen for determining the scale factors in equation (1) contains 1794 molecule/ion pairs extracted from 171 organic, inorganic and metal–organic molecular crystal structures, including atoms up to Br, as well as I and Xe. For each crystal structure, molecule/ion pairs were obtained by generating a cluster of nearest neighbours surrounding each unique molecule/ion in the structure. In addition to the original set of 232 neutral pairs, this contains 751 pairs from organic and metal–organic salts, 583 pairs from neutral closed-shell metal organic crystal structures and 228 pairs incorporating open-shell species. In addition to a wide range of interactions between neutral organic and inorganic molecules, these four broad categories encompass large numbers of pairs involving ion–ion and ion–solvate/hydrate interactions, as well as neutral and ionic metal coordination compounds, including solvates. We believe the training set is well balanced (in terms of representation of a very wide range of atoms and interaction types) and sufficiently robust that removal or addition of a small number of structures has a minimal effect on the outcomes. *Mercury* (Macrae *et al.*, 2008 ▸) was used to add H atoms for a small number of structures and, as before, all *X*—H covalent bond lengths were normalized to standard values from neutron diffraction (Allen *et al.*, 2004 ▸). The supporting information provides details of all crystal structures, including CSD refcodes or ICSD identifiers, compound names, molecular diagrams and references to the crystal structure determinations.

### 2.2. Modifications to the *CrystalExplorer* energy models and frameworks   

As in our original publication (Turner *et al.*, 2014 ▸), the two energy models described here are based on unperturbed electron distributions computed at either B3LYP or Hartree–Fock levels of theory. As before, in the CE-B3LYP model, the 6-31G(d,p) basis set is used for molecules containing atoms H to Kr, and for species containing heavier atoms, the DGDZVP basis set has been used. The faster and less accurate CE-HF model uses the 3-21G basis set for all atoms. Because computation of MP2 molecular wavefunctions is more time consuming than with the B3LYP functional, and the model energies not obviously superior, we did not pursue any further the CE-MP2 model energy that was based on MP2/6-31G(d,p) monomer electron densities.

#### 2.2.1. Polarizabilities for monatomic cations and anions   

In applying our original energy model to salts it became obvious that the energies for pairs involving monatomic cations were far too large, due to the use of atomic polarizabilities for calculating polarization energies involving these species. Table 1 ▸ lists the polarizabilities for neutral atoms from Thakkar & Lupinetti (2006 ▸), along with values for monatomic cations and anions selected from the literature; the latter values are used in all calculations reported in this work. The values for cations are smaller than those for atoms by orders of magnitude. Polarizabilities for the halide anions are larger than the atomic values, but the difference is much smaller than for cations. We emphasize that these polarizabilities are only used when the relevant atomic species is clearly and unambiguously a monatomic ion, and not covalently bound.

#### 2.2.2. Open-shell systems   

In our original publication, we restricted the training set of crystals to neutral closed-shell molecules and noted that the exchange–repulsion energy in our model was calculated from the antisymmetric product of the monomer spin orbitals. Extension to open-shell unrestricted Hartre–Fock (UHF) wavefunctions was undertaken in the manner described by Su & Li (2009 ▸). Although the spin state of the dimer is, in principle, undefined, we have assumed in all cases that the spin multiplicity of the dimer reflected two unpaired spins, one from each of the monomers. In applying these methods to open-shell metal coordination compounds, it is of course essential to calculate monomer wavefunctions with a spin multiplicity appropriate to the oxidation state of the metal, and for systems with odd numbers of electrons this was always taken to be a doublet UHF state. For calculation of benchmark energies, the B3LYP-D2 counterpoise-corrected energy also had to reflect the assumption of unpaired spins for the dimer [*e.g.* for two monomer species, each with one unpaired electron (doublets), the state of the dimer was always chosen to be the high-spin UHF triplet].

#### 2.2.3. Destabilizing terms in energy frameworks   

The original implementation of energy frameworks was restricted to electrostatic and dispersion-energy terms and total energies of negative sign, implicitly assuming that these stabilizing energies are most relevant to discussing the crystal structures of neutral molecules. However, ionic crystals necessarily incorporate large positive destabilizing (cation–cation and anion–anion), as well as large negative (cation–anion) energies, and these need to be represented as part of an energy-framework picture. To this end, we have added cylinders of a different colour (yellow in the examples presented here) to energy-framework diagrams for the electrostatic term (red) and total energy (blue).

### 2.3. Benchmark DFT calculations   

*GAUSSIAN09* (Frisch *et al.*, 2009 ▸) was used to determine all pairwise intermolecular/interionic energies for calibration purposes, based on B3LYP-D2 calculations corrected for BSSE. For a very small number of crystal structures involving anion–cation pairs, supermolecule calculations at this level did not converge and these ion–ion pairs were not included in the fitting process, although energies for all other possible molecule/ion pairs in those structures were included. HF/3-21G monomer calculations also failed to converge for some open-shell molecules/ions [Cambridge Structural Database (CSD; Groom *et al*., 2016 ▸) refcodes ACACCR07, ACACVO04, CPNDYV07, IGACEC, JIYKEH, AFATAE and AGEFEX], and those structures were not included in the determination of scale factors for the CE-HF energy model. Perhaps of more consequence, the B3LYP-D2 counterpoise-corrected benchmark calculations provided quite obviously unacceptable energies for the crystalline salts ferrocenium tris­(hexa­fluoro­acetylacetonato)manganese(II) (AGEFEX), the 1,2-di­phenyl­ethylenediammonium *N*-phenyliminodiacetate ethanol sol­vate (KOLDUL), calcium α-ethylmalonate (CUZHEK) and sodium dihydrogen citrate (NAHCIT). For these crystal structures, the B3LYP-D2 counterpoise-cor­rected energies between pairs of ions were consistently much more binding than obtained with a simple electrostatic model; MP2/6-31G(d,p) counterpoise-corrected calculations were used as benchmark energies instead.

## 3. Results and discussion   

### 3.1. Overall fitting results   

**Figure 1:**
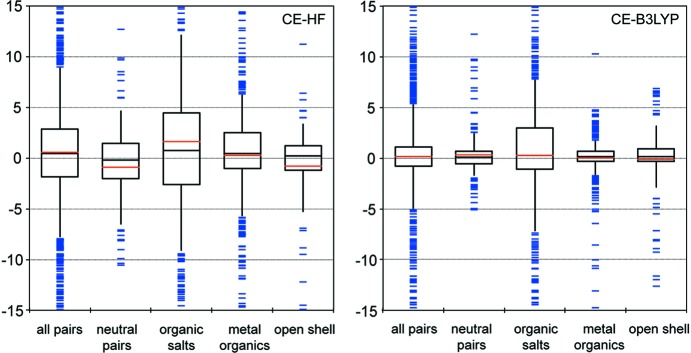
Box-and-whisker plots summarizing the deviations of CE-HF and CE-B3LYP model energies from benchmark values. The vertical axis is in kJ mol^−1^, and for each model separate box plots are provided for results across all molecule/ion pairs, as well as separate subsets of neutral pairs, organic salts, metal coordination compounds and open-shell systems. Each box contains the interquartile range (*i.e.* the middle half of the data), the median value is indicated by a black line, the mean value is indicated by a red line and the whiskers extend one standard deviation from the mean. Individual outliers (*i.e.* deviations beyond the mean ± one standard deviation) are indicated by blue bars. For clarity, the plots depict only deviations in the range ±15 kJ mol^−1^.

Scale factors and fit statistics for CE-HF and CE-B3LYP model energies, as well as mean absolute (MAD), mean (MD), root-mean-square (RMSD) and minimum and maximum deviations from benchmark energies, are summarized in Tables 2 ▸ and 3 ▸. Fig. 1 ▸ provides a complementary graphical depiction of the deviations in the form of box-and-whisker plots, and it is important to recognize that for each of the two models, a single fit has been performed to obtain the four scale factors in equation (1), and data relevant to that fit for all molecule/ion pairs are labelled ‘all pairs’. Statistics obtained by applying those fitted scale factors to separate subsets of molecule/ion pairs are labelled ‘neutral pairs’ (*i.e.* the same set upon which our earlier fitting was based), ‘organic salts’, ‘metal–organics’ and ‘open shell’. As reported in our previous work, the CE-HF model is clearly inferior to CE-B3LYP – and for good reason – but it nevertheless performs remarkably well, with an overall MAD of only 4.7 kJ mol^−1^. We only recommend this model for situations where a quick and approximate set of energies is required, or for applications to very large systems, and focus the remaining discussion in this section on the more accurate and reliable CE-B3LYP model.

The revised scale factors for the CE-B3LYP model differ only slightly from those reported earlier, a consequence of the interplay between the four energy terms, as seen in the correlation coefficients listed in Table 3 ▸. We note that the polarization and electrostatic energies are weakly positively correlated and the repulsion energy is weakly negatively correlated, with the other three terms and the dispersion energy showing no correlation at all with electrostatic or polarization terms – it is clearly describing a distinctly separate phenomenon. One very pleasing outcome from the present fit to a much wider range of interactions is that the model energies obtained for the previous molecular pairs (‘neutral pairs’) are only marginally different from those obtained previously; the MAD between old and new CE-B3LYP model energies for this subset of 232 neutral pairs is 0.4 kJ mol^−1^.

The box plots in Fig. 1 ▸ are instructive, as they highlight not only the very clear differences between the CE-HF and CE-B3LYP models, but also the performance of the models for different subsets of molecule/ion pairs. CE-B3LYP model energies more accurately model benchmark results, and with similarly small deviations, for ‘neutral pairs’, ‘metal–organics’ and ‘open-shell’ systems – MAD values are less than 2 kJ mol^−1^ for all three subsets (Table 3 ▸) – while the deviations for ‘organic salts’ are considerably larger. Similar comparisons are evident in the distributions of outliers for each subset. These broad conclusions are unsurprising, as the range of benchmark B3LYP-D2 counterpoise-corrected energies is 328 kJ mol^−1^ for ‘neutral pairs’, 210 kJ mol^−1^ for ‘metal–organics’, 520 kJ mol^−1^ for ‘open shell’, but 3751 kJ mol^−1^ – an order of magnitude greater – for ‘organic salts’.

As described in §2.1, the crystal structures used to construct the training set of molecule/ion pairs included atoms up to Br, as well as I and Xe; we deliberately omitted metal coordination compounds incorporating metals of the second transition series, *i.e.* Y to Cd. After the fitting was complete, we used the final scale factors and equation (1) to estimate the intermolecular energies for 100 molecular pairs extracted from 13 metal–organic crystal structures incorporating Zr (CSD refcodes DAQFEH, PIPNEJ and CCPZRA), Mo (KUJLEG, JEVKAW and FUBYIK01), Tc (KABMIJ and KITDAS), Ru (CYCPRU09, FALZAV and ACACRU03), Rh (ACACRH10) and Pd (DETCPD01). For this purpose, B3LYP monomer wavefunctions were calculated using the DGDZVP basis set on all atoms, and the resulting CE-B3LYP energies compared with counterpoise-corrected B3LYP-D2 interaction energies computed with a mixed basis set, *i.e.* DGDZVP on second-row transition metals and 6-31G(d,p) on all other atoms. The resulting mean absolute error between the CE-B3LYP model energy estimates and benchmark DFT energies was only 0.7 kJ mol^−1^, indicating that not only are these model energies applicable to systems including atoms up to Xe (and probably beyond), but also that the DGDZVP and 6-31G(d,p) basis sets yield monomer wavefunctions of very similar quality and are essentially interchangeable for our purposes.

## 4. Examples   

In this section, we summarize results for CE-B3LYP model energies and energy frameworks applied to a small number of crystals, for comparison with other recent results, but also to highlight the insight into molecular crystal structure that can be derived in this manner.

### 4.1. Chromium hexacarbonyl   

**Figure 2:**
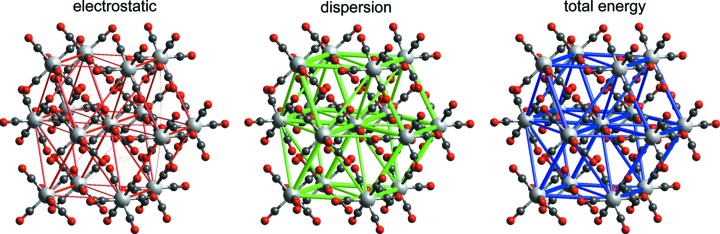
Energy-framework diagrams for *E*_ele_, *E*_dis_ and *E*_tot_ for a cluster of nearest-neighbour molecules in Cr(CO)_6_ (CSD refcode FOHCOU02). All diagrams use the same cylinder scale of 150 for energies.

Maloney *et al.* (2015 ▸) reported results for chromium hexa­carbonyl (CSD refcode FOHCOU02) as an example in their extension of the PIXEL method to metal coordination com­pounds. Table 4 ▸ compares those results for close intermolecular contacts with the CE-B3LYP values. The elec­tro­static terms are essentially identical, as expected, but the PIXEL dispersion and repulsion components are all greater than those from the CE-B3LYP model, by factors of approximately 1.3 and 1.5, respectively; polarization terms are too small for a useful comparison. Total energies are more similar, with PIXEL values larger than CE-B3LYP by ∼15%, and this difference is reflected in the lattice energies obtained by summation of pairwise energies to convergence: −71 (PIXEL) *versus* −63 kJ mol^−1^ (CE-B3LYP), both comparing favourably with the median value of −69.4 kJ mol^−1^ from a large number of experimental sublimation enthalpies (Acree & Chickos, 2016 ▸). We note that the PIXEL result is based on optimization of parameters to fit experimental sublimation enthalpies, whereas the CE-B3LYP result is based on a fit to DFT energies. (We quote all CE-B3LYP results as whole numbers with the expectation that individual pairwise energies are certainly less reliable than 1 kJ mol^−1^.) As observed by Maloney *et al.* (2015 ▸), these energies confirm ‘that the interactions are predominantly dispersion based’, and this is revealed clearly by the energy-framework diagrams in Fig. 2 ▸, where the magnitude of the dispersion energies closely mirrors the total energies; the electrostatic term is not insignificant, but largely cancelled by repulsion in each case.

### 4.2. Bis(acetylacetonato)oxidovanadium(IV), VO(acac)_2_   

**Figure 3:**
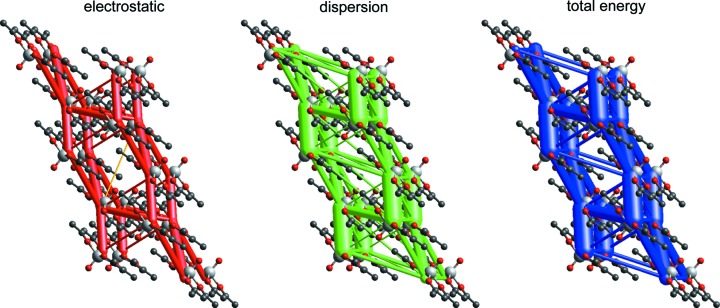
Energy-framework diagrams for *E*_ele_, *E*_dis_ and *E*_tot_ for a cluster of molecules in VO(acac)_2_ (CSD refcode ACACVO12). H atoms have been omitted for clarity and all diagrams use the same cylinder scale of 150 for energies.

For this compound, Maloney *et al.* (2015 ▸) identified the stacking interaction between molecules across an inversion centre as the strongest intermolecular contact, with a PIXEL-derived energy of −65.0 kJ mol^−1^, and this agrees with the CE-B3LYP result of −68 kJ mol^−1^. Individual energy com­ponents are also in closer agreement in this case. Energy frameworks for this structure (Fig. 3 ▸) clearly show the strong stacking interaction as vertical cylinders, and these are linked within and between planes by obviously important interactions of lesser strength. Both Figs. 2 ▸ and 3 ▸ depict energies on the same scale relative to molecular dimensions, and it is readily seen that the intermolecular interactions in Cr(CO)_6_ are substantially weaker, and more isotropic in nature, than those in VO(acac)_2_. The PIXEL estimate for lattice energy of −143.7 kJ mol^−1^ compares favourably with the mean sublimation enthalpy of 140.6 (4) kJ mol^−1^. The CE-B3LYP estimate of −153 kJ mol^−1^ is somewhat greater, but again we emphasize that this represents a converged sum over 41 different pairwise interactions, for which the largest individual pairwise energies are (in descending order) −67.7, −49.8, −41.9, −31.3, −25.1, −12.7, −11.4 and −5.8 kJ mol^−1^, all of which will have an inherent uncertainty of *at least* 1 kJ mol^−1^ based on the MAD for this subset in Table 3 ▸.

### 4.3. Ferrocene ‘dimers’ in 1,1′-dimethylferrocene?   

**Figure 4:**
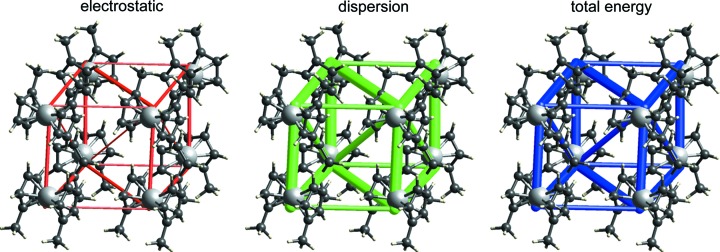
Energy-framework diagrams for *E*_ele_, *E*_dis_ and *E*_tot_ for a cluster of molecules in 1,1′-di­methylferrocene (CSD refcode ZAYDUY02). The ferrocene ‘dimer’ proposed by Bogdanović & Novaković (2011 ▸) is the vertical pair of molecules, with an Fe⋯Fe distance of 5.709 Å. All diagrams use the same cylinder scale of 150 for energies.

Based on a detailed analysis of structures in the CSD which revealed that nearly 47% of ferrocene crystal structures include a side-by-side pairing of parallel ferrocene molecules displaced by one-half the distance between the cyclopentadienyl (Cp) ring centroids, Bogdanović & Novaković (2011 ▸) pro­posed this particular rigid ferrocene–ferrocene dimer as a common ‘building block’ in the crystal structures of ferrocenes. Additional evidence for this conclusion came from an analysis of molecular electrostatic potentials derived from an earlier experimental charge–density analysis of 1,1′-di­methyl­ferrocene (Makal *et al.*, 2010 ▸). CE-B3LYP energy frameworks for 1,1′-di­methyl­ferrocene (Fig. 4 ▸) show no evidence of an identifiable dimer in this structure. Instead, the total-energy-framework diagram reveals a relatively isotropic topology of intermolecular interactions, each molecule being involved in six relatively strong interactions, and where the dominant component is clearly dispersion. Electrostatic energies are all less than 9 kJ mol^−1^ in magnitude, whereas dispersion energies are as strong as −27 kJ mol^−1^. The proposed ferrocene ‘dimer’ in this crystal structure is certainly stabilizing, with a total interaction energy of −22 kJ mol^−1^, but there are three additional molecular pairs with similar energies (−21, −21 and −23 kJ mol^−1^), strongly suggesting that the ‘dimer’ proposed by Bogdanovic & Novakovic is not special, and definitely not a ‘building block’ in any sense. There is an important lesson here: the common occurrence of a particular structural motif does not necessarily imply that it is structure determining or an important supramolecular synthon, and the energy-framework diagram can readily provide insight into its true nature.

### 4.4. Glycine crystal structures and destabilizing energies in energy frameworks   

**Figure 5:**
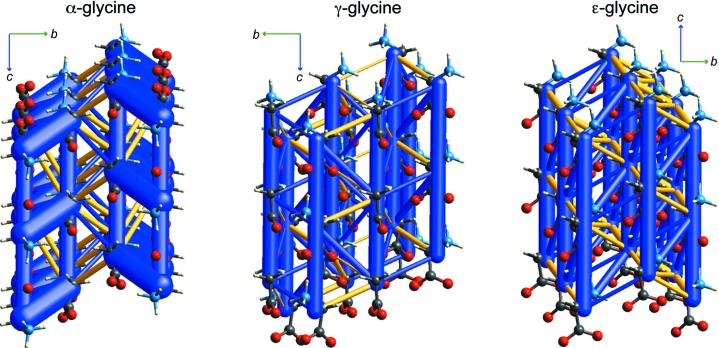
Total-energy-framework diagrams for three glycine polymorphs. All diagrams use the same cylinder scale of 50 for energies (*i.e.* one third of that used in Figs. 2 ▸, 3 ▸ and 4 ▸) and energies with magnitude less than 15 kJ mol^−1^ have been omitted. CE-B3LYP energies were calculated for room-temperature structures determined by neutron diffraction in all cases (left to right: CSD refcodes GLYCIN19, GLYCIN16 and DOLBIR14).

In the *Introduction* (§1), we noted the work of Moggach *et al.* (2015 ▸) on the high-pressure polymorph ∊-glycine, which made particular note of the numerous destabilizing molecule–molecule interactions in ∊-glycine, with PIXEL estimates of intermolecular energies as large as +51 kJ mol^−1^. Destabilizing interactions of this kind in crystal structures of glycine and other amino acids are not unusual (Destro *et al.*, 2000 ▸; Gavezzotti, 2002 ▸; Dunitz & Gavezzotti, 2012 ▸). Here we use energy frameworks to visualize the relative magnitude and topology of the positive interaction energies for three different glycine polymorphs, *i.e.* α, γ and ∊ (Fig. 5 ▸).

The crystal structure of α-glycine has been described as comprising antiparallel double layers in the *ac* plane (Langan *et al.*, 2002 ▸), and these are seen clearly in Fig. 5 ▸, with molecules in these double layers involved in three distinct strong hydrogen-bonded interactions: −177 (cyclic dimer), −124 (also a cyclic dimer) and −106 kJ mol^−1^ (head-to-tail along *c*, single hydrogen bond). The double layer also includes a significant destabilizing interaction of +37 kJ mol^−1^. The energy framework also clearly shows that these double layers are relatively weakly bound to one another along *b*, with nearest-neighbour interactions betweeen molecules in adjacent layers of −31 and +40 kJ mol^−1^. This anisotropy of intermolecular interactions correlates nicely with the observed anisotropic thermal expansion between 100 and 400 K, where the relative increase in *b* is far greater than for *a* or *c* (Langan *et al.*, 2002 ▸). The topology of the energy framework in Fig. 5 ▸ is also entirely consistent with recent measurements of Young’s modulus for α-glycine based on nanoindentation experiments (Azuri *et al.*, 2015 ▸). The smallest value of 26±1 GPa was measured for the (010) face (perpendicular to *b*), and the largest value of 44±1 GPa was measured for the (001) face, perpendicular to *c*.

Another stable form of glycine at ambient temperature and pressure, apparently more stable than α-glycine (Perlovich *et al.*, 2001 ▸), is γ-glycine. This structure has been described as ‘consisting of glycine molecules linked by two hydrogen bonds (N—H1⋯O1 and N—H2⋯O2) to form helices around the crystallographic 3_2_ screw axes. A third lateral hydrogen bond (N—H3⋯O1) connects the helices, thus forming a three-dimensional network’ (Kvick *et al.*, 1980 ▸). Fig. 5 ▸ shows that the strongest interaction is clearly the head-to-tail N—H1⋯O1 arrangement along *c* (−106 kJ mol^−1^). The N—H2⋯O2 hydrogen bond creates the helical motif, but is much weaker at −32 kJ mol^−1^, and the N—H3⋯O1 link between helices is actually destabilizing at +18 kJ mol^−1^. The strongest interaction between helices is actually largely dipole–dipole, between molecules displaced sideways and along *c* (−48 kJ mol^−1^). We note that for both polymorphs stable at ambient temperature and pressure, the strongest detabilizing interactions evident in Fig. 5 ▸ are similar in magnitude and frequency (+38 and +18 kJ mol^−1^ in γ-glycine, and +40, +37 and +17 kJ mol^−1^ in α-glycine).

**Figure 6:**
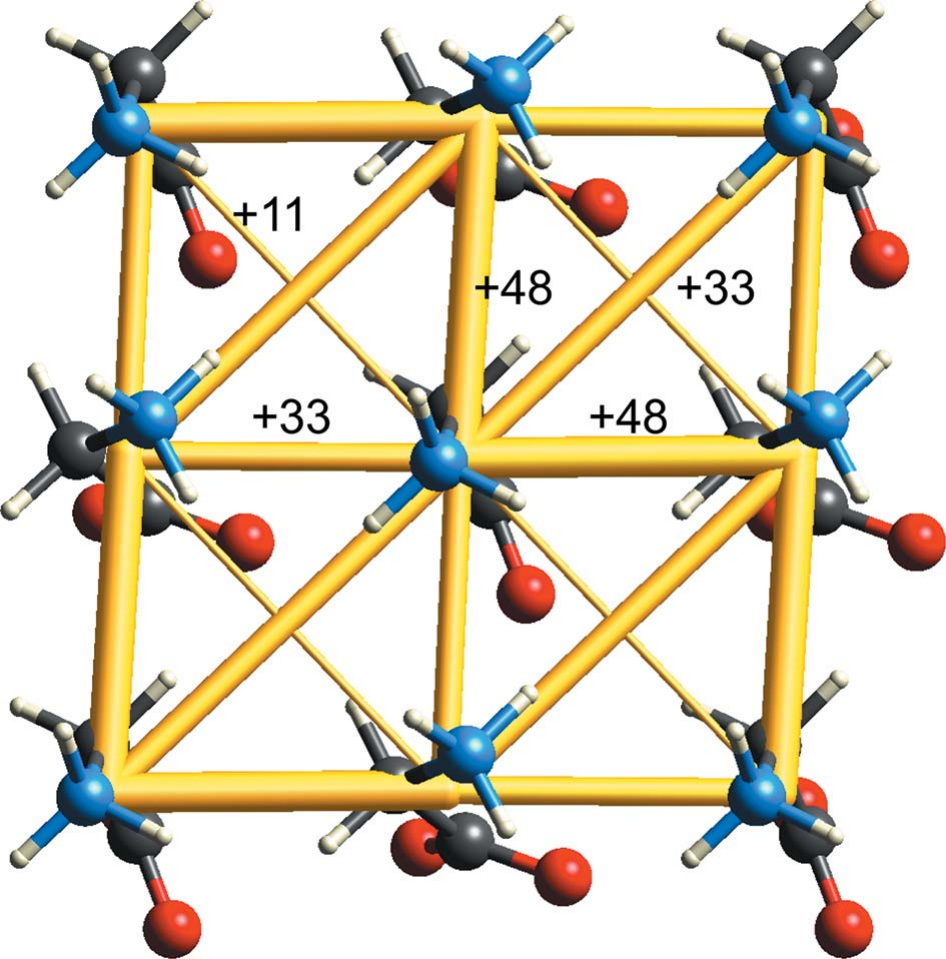
Energy-framework diagram for destabilizing interactions in ∊-glycine (CSD refcode DOLBIR14). The cylinder scale is the same as in Fig. 5 ▸ and energies for all nearest-neighbour pairs are included (kJ mol^−1^).

The dominant feature of head-to-tail chains of molecules along *b* is also evident in the high-pressure polymorph, *i.e.* ∊-glycine, studied in detail by Moggach *et al.* (2015 ▸). In this structure, this hydrogen bond has essentially the same energy as in the other two polymorphs (−107 kJ mol^−1^), and these chains are linked by interactions of −58 and −43 kJ mol^−1^. However, Fig. 5 ▸ also reveals prominent networks of destabilizing energies, all of which occur in what might be termed a ‘layer’ of molecules with all dipoles aligned (seen on the diagonal in Fig. 4 ▸). The energies for these destabilizing interactions are +48 and +33 kJ mol^−1^, arising almost entirely from the electrostatic interaction between dipolar molecules arranged in parallel side-by-side. These interactions are seen more clearly in Fig. 6 ▸, which highlights how all nearest-neighbour interactions (and presumably even longer-range interactions) in this ‘layer’ are in fact destabilizing. Although individual pairwise interactions were discussed by Moggach *et al.* (and with PIXEL-derived energies very similar to the present CE-B3LYP ones), we see here how energy frameworks can clearly reveal features of the packing of molecules in a crystal that are otherwise hidden.

In addition to total PIXEL estimates of interaction energies for seven nearest neighbours in ∊-glycine, Moggach *et al.* also reported the individual components of those energies (Table 2 ▸ in that work), and it is instructive to compare those with the present CE-B3LYP results. There is excellent agreement for the total and electrostatic energies, with a mean absolute deviation between the two models of only 1.8 kJ mol^−1^; CE-B3LYP total energies are on average 99% of those from PIXEL, while electrostatic energies are 98% of PIXEL values. But there are large differences between the two schemes for polarization, dispersion and repulsion energies: CE-B3LYP energy components are typically 61% (polarization), 71% (dispersion) and 60% (repulsion) of those from PIXEL. This is unsurprising, given the arbitrary nature of the subdivision in equation (1), and the completely different origin of each of those energy terms in the two schemes, but the fact that these three ratios are not far from the optimum CE-B3LYP scale factors *k*_pol_ (0.740), *k*_dis_ (0.871) and *k*_rep_ (0.618) (Table 3 ▸) hints at a deeper relationship between the PIXEL and *CrystalExplorer* interaction-energy terms.^1^ We find it remarkable that the total interaction energies agree so well for these molecular pairs, but emphasize that the very large differences between the PIXEL and CE-B3LYP polarization, dispersion and repulsion components mean that it cannot be particularly productive or meaningful to discuss or compare the absolute values of these terms, although valid comparisons can certainly be made within one or other of these schemes.

### 4.5. Interactions between ions: adducts of pyridine and formic acid   

**Figure 7:**
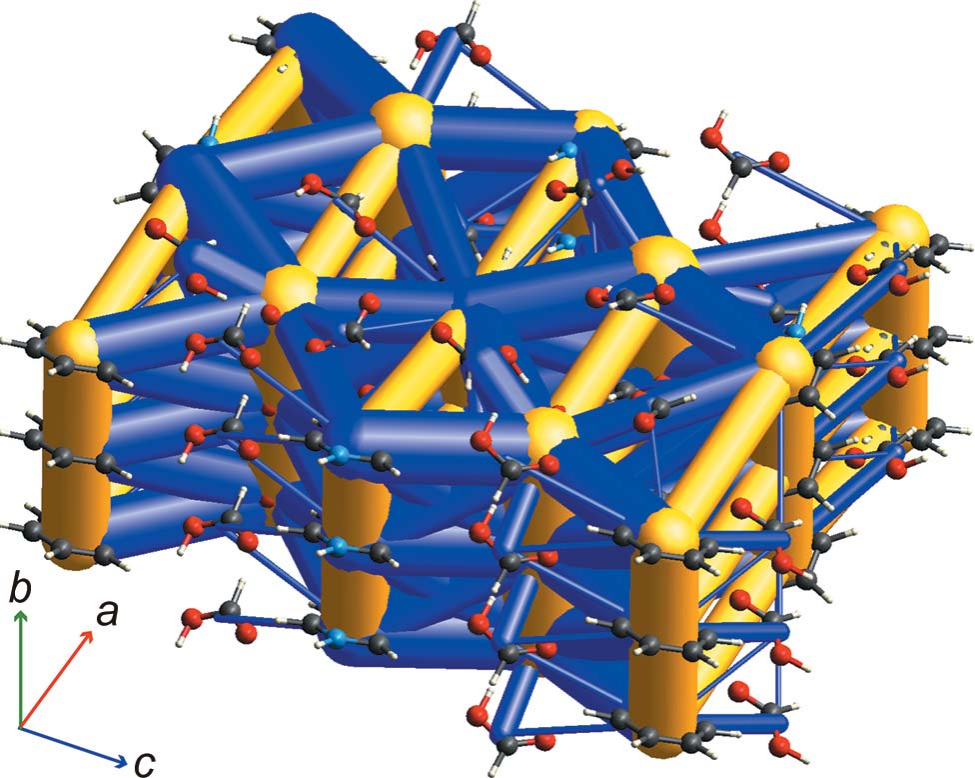
Energy-framework diagram for pyridinium formate tris­(formic acid) (CSD refcode QAFFOS). A cylinder scale of 40 is used in this case to enable representation of ion–ion energies, as well as interactions between neutral species, and energies with a magnitude less than 20 kJ mol^−1^ have been omitted. The cluster displays contents of three unit cells along *b*.

Crystal structures of two adducts of pyridine and formic acid have been reported by Wiechert & Mootz (1999 ▸), one a cocrystal with a 1:1 stoichiometry consisting of neutral molecules, and the other a salt with a 1:4 stoichiometry, namely pyridinium formate tris­(formic acid). Both are low-melting-point solids, with melting points of 219 and 233 K, respectively. The structure of the cocrystal is straightforward to describe, being dominated by the O—H⋯N hydrogen-bonded pyridine–formic acid heterodimer with energy −53 kJ mol^−1^; the next largest interactions are −13 (between formic acid molecules along the 2_1_ axis) and −7 kJ mol^−1^ (a weaker C—H⋯O heterodimer interaction). Interaction energies between ions in the pyridinium salt are much greater, and Fig. 7 ▸ illustrates how an energy-framework diagram can shed some light on the various interactions in that structure. Ion–ion interactions are, of course, long range and it needs to be recognized that the interactions depicted in Fig. 7 ▸ are only those between molecules in relatively close proximity (but further than nearest neighbours in this case); there are many many more interactions at longer separations. Fig. 7 ▸ shows a very strong stabilizing pyridinium–formate interaction in the *ac* plane (−253 kJ mol^−1^) and another between ions in adjacent planes (−199 kJ mol^−1^). Less strong are formate–formic acid interactions (−104 and −115 kJ mol^−1^), while the strongest pyridinium–formic acid interaction is −80 kJ mol^−1^. These sta­bilizing interactions are counterbalanced by some even stronger destabilizing interactions between adjacent formate anions (+342 kJ mol^−1^) and pyridinium cations (+268 kJ mol^−1^) ‘stacked’ along *b*, and between formate anions (+184 kJ mol^−1^) and pyridinium cations (+182 kJ mol^−1^) along *a*. The latter energies, between molecular ions of the same charge separated by *a*/2 (8.175 Å), approach the energy between unit charges at that separation, *i.e.* +169 kJ mol^−1^. Although the topology of stabilizing and destabilizing interactions depicted in Fig. 7 ▸ is complex, it is worth emphasizing that it is also incomplete, as interactions smaller than ±20 kJ mol^−1^ have been omitted for clarity, but also because numerous strong interactions between ions in that cluster have not even been included in the calculation of pairwise energies. They could have been included, but the result would have been an almost indecipherable energy framework. This highlights an inherent limitation of energy frameworks – and it is suggested by the word ‘framework’. Because they conveniently represent interactions between nearest-neighbour moieties, they are well suited to crystals where the interaction energy is relatively short range (*e.g.* between molecules with zero or small dipole moments), less ideal for highly polar molecules (*e.g.* amino acids) and should be used with caution when applied to ionic crystals.

### 4.6. Open-shell molecules: a nitro­nyl nitroxide organic free radical   

**Figure 8:**
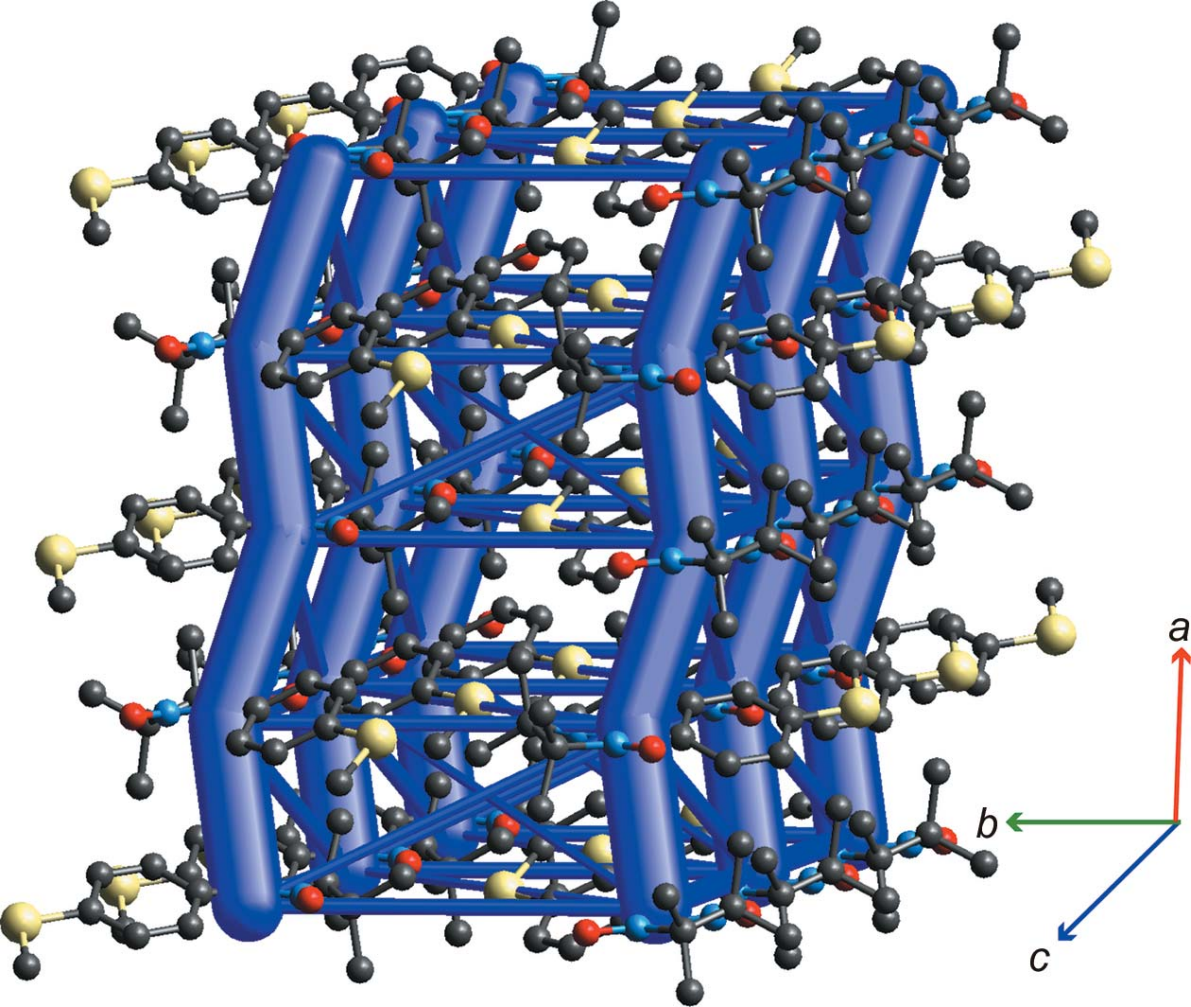
Energy-framework diagram for *p*-(methylthio)phenyl nitronyl nitroxide at 114 K (CSD refcode YUJNEW11). H atoms have been omitted for clarity and a cylinder scale of 150 is used in this case; energies with a magnitude less than 7 kJ mol^−1^ have been omitted. The cluster displays the contents of two unit cells along *a*.

Nitronyl nitroxide free radicals have been the subject of studies focusing on the mechanism of magnetic interactions in the crystals, as their bulk magnetic behaviour is known to be very sensitive to both the chemical structure of the spin carrier, as well as the crystal packing. *p*-(Methylthio)phenyl nitronyl nitroxide [Nit(SMe)Ph, 2-(4-methylthiophenyl)-4,4,5,5-tetramethylimidazoline-1-oxyl-3-oxide], perhaps the most studied of this family, is ferromagnetic below 0.2 K, and has been investigated by polarized neutron diffraction (Pontillon *et al.*, 1999 ▸), experimental charge–density analysis (Pillet *et al.*, 2001 ▸), as well as a detailed theoretical study that determined its magnetic topology by calculating magnetic interactions (*J*_AB_ exchange couplings) between key radical pairs in the crystal (Deumal *et al.*, 2004 ▸). Fig. 8 ▸ presents the energy-framework diagram for this organic radical, based on the crystal structure determined at 114 K (CSD refcode YUJNEW11). The orientation of the molecular cluster has been chosen to closely replicate that in Fig. 3 ▸(*c*) of Deumal *et al.* (2004 ▸), which illustrated the magnetic topology based on computed values of four key magnetic pair interactions. Although there is an obvious similarity between the two diagrams, the interaction energies between adjacent molecules are tens of kJ mol^−1^, while the computed exchange couplings are only a fraction of a cm^–1^; Table 5 ▸ summarizes these results for the nine radical pairs identified by Deumal *et al.* (2004 ▸).

**Figure 9:**
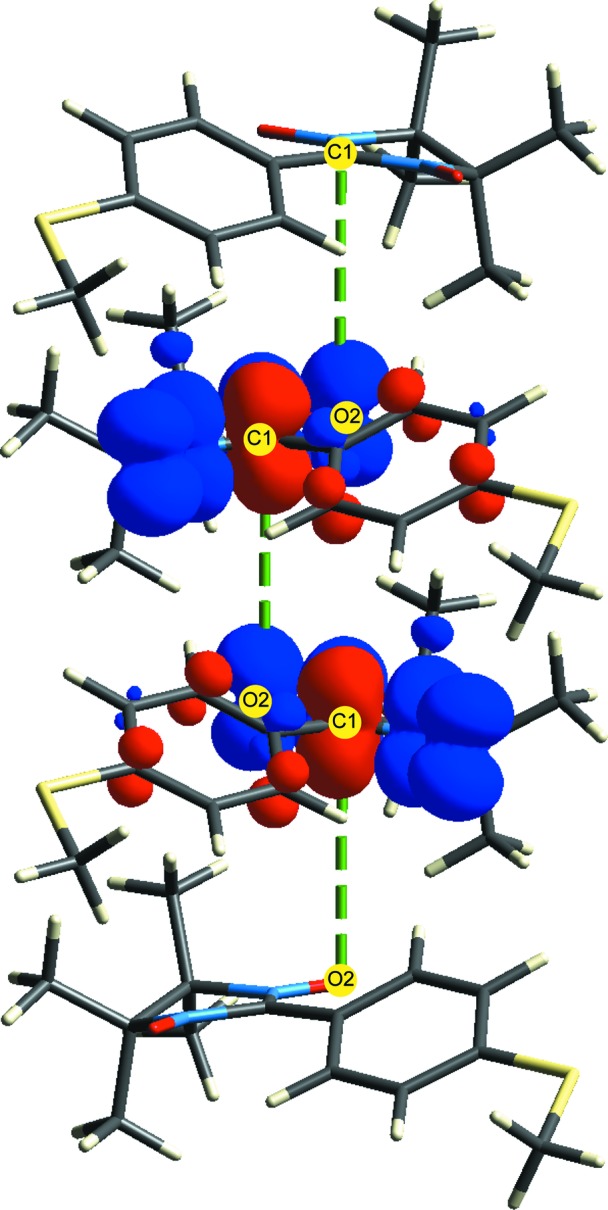
Spin-density isosurfaces for *p*-(methylthio)phenyl nitronyl nitroxide from a UB3LYP/6-31G(d,p) wavefunction for the doublet state. Isosurfaces are at ±0.002 au; blue corresponds to positive spin density and red to negative. The spin density is largely localized on the O—N—C—N—O moiety, and the cluster displays the d1 radical pairing stacked along *a*. Green dashed lines highlight the C1⋯O2 separation mentioned in the text.

Three of those radical pairs (d1, d2 and d3) link molecules in the *ac* plane, while the remaining six link molecules between adjacent *ac* planes. Deumal *et al.* concluded that the magnetic topology based on the 298 K structure is two-dimensional, with the largest exchange couplings being *J*_AB_(d1), *J*_AB_(d2) and *J*_AB_(d3) (Table 5 ▸). For lower-temperature structures, *J*_AB_(d6) is larger and *J*_AB_(d3) is smaller than for the room-temperature structure, resulting in a three-dimensional magnetic topology. Is there a correlation between changes in CE-B3LYP energies and *J*_AB_ values for these three crystal structures? From Table 5 ▸ we see that CE-B3LYP radical pair interaction energies show little change with temperature, with the exception of those for d1 and d6, which are both significantly stronger at the lower temperatures. The key factor in the change from a two-dimensional to a three-dimensional magnetic structure is the substantial increase in *J*_AB_(d6). This does correlate with the strengthening of the intermolecular energy for this pair, but it needs to be emphasized that this does not mean that *J*_AB_ depends on the interaction energy. The enhancement of both the intermolecular and magnetic interactions is a consequence of the shrinking of the unit cell with decreasing temperature; the Me—S⋯O—N distance falls from 4.20 (298 K) to 3.95 Å (10 K), and the almost coplanar methyl-H⋯O—N separation falls from 3.11 (298 K) to 2.78 Å (10 K). The strongest intermolecular interaction is d1, and this also exhibits the largest exchange coupling at all temperatures. This interaction corresponds to pairs of molecules related by the twofold screw axis along *b*, stacked along *a*, with overlap betweeen the phenyl and nitronyl nitroxide moieties of adjacent radicals. With decreasing temperature, the O2⋯C1 separation falls from 4.25 to 4.17 Å, and it is perhaps not incidental that this stacking motif in the crystal coincides with a very obvious chain of overlapping spin densities between adjacent molecules, propagating along *a* (Fig. 9 ▸).

## 5. Summary and outlook   

This research has been motivated by the conviction that a whole-of-molecule approach is essential if we are to fully understand the nature of intermolecular interactions in the context of crystal packing. Such an approach avoids a focus on specific atom–atom interactions, or what appear to be novel interactions, which can lead to the neglect of others that may be more energetically important. In this and other recent work (Edwards *et al.*, 2017 ▸), we have outlined one way of achieving this, *i.e.* using the graphical and computational tools embodied in our research toolbox *CrystalExplorer*, but it is certainly not the only one. The broad details of noncovalent interactions can be largely understood through their common origin in the redistribution of electron density upon bonding, which leads directly to the molecular electrostatic potential and qualitative concepts, such as electrostatic complementarity, and from there to the efficient calculation of reliable intermolecular interaction energies. Visualization of these energies and their electrostatic and dispersion components, in the form of energy frameworks, sheds light on the architecture of molecular crystals, in turn providing a potential link to actual crystal properties.

Here we have built on our earlier work (Turner *et al.*, 2014 ▸) to considerably expand the realm of application of CE-HF and CE-B3LYP model energies to include neutral organic molecules (including other *p*-block elements), metal coordination compounds, organic salts, solvates and radicals. The new sets of scale factors that have been determined by fitting to counterpoise-corrected DFT calculations result in minimal changes from previous energy values but, along with the use of separate polarizabilities for interactions involving monatomic ions, they mean that the *CrystalExplorer* model energies can now be applied with confidence to a vast number of molecular crystals. The mean absolute deviation of these model energies from benchmark DFT results is 4.7 kJ mol^−1^ for HF/3-21G electron densities and as little as 2.5 kJ mol^−1^ when B3LYP/6-31G(d,p) monomer electron densities are used. Given the magnitude of these deviations, we recommend always reporting model energies as whole numbers in kJ mol^−1^.

The earlier implementation of energy frameworks (Turner *et al.*, 2015 ▸) now incorporates additional cylinders to represent destabilizing interactions (*i.e.* those with positive energies), an enhancement that is important for molecules exhibiting large dipole moments (such as zwitterions) and, of course, organic salts (although it is worth noting that energy frameworks are potentially of less value when exploring interactions in crystals involving charged species). The energy models and other enhancements described in this work have all been implemented in *CrystalExplorer17* (Turner *et al.*, 2017 ▸), now released and freely available to academic users.

Through applications to a variety of interesting molecular crystals, we have attempted to provide examples of how these new computational and graphical tools may be used to enhance the understanding of the nature of intermolecular interactions in the context of crystal packing, and to highlight the utility and promise of these tools. We anticipate that the ideas and approaches presented here will find widespread application, and we are currently exploring their use in estimating lattice energies for crystals comprised of neutral molecules, as well as investigating a possible link between energy frameworks and the mechanical properties of molecular crystals.

## Supplementary Material

Crystal structure details (ICSD numbers, CSD refcodes, compound names, structure diagrams and references) for all structures used to derive molecule/ion pairs.. DOI: 10.1107/S205225251700848X/lc5090sup1.pdf

## Figures and Tables

**Table 1:** Isotropic polarizabilities (atomic units) chosen for monatomic cations and anions For comparison with the values chosen for monatomic ions, the first row for each atomic species also lists the atomic polarizability (Thakkar & Lupinetti, 2006 ▸).

	Li	Na	K	Rb	Cs
Neutral atom	164.0	162.9	291.1	316.2	396.0
Cation, *X* ^+^	0.190†	0.986†	5.4†	9.1†	15.7†
					
	Be	Mg	Ca	Sr	Ba
Neutral atom	37.74	71.22	157.9	199.0	273.5
Cation, *X* ^2+^	0.052†	0.482†	3.2†	5.8†	10.6†
					
	F	Cl	Br	I	
Neutral atom	3.70	14.57	21.13	32.98	
Anion, *X* ^−^	7.25‡	21.2‡	27.9§	39.6¶	

† Average of theoretical and experimental values (Mitroy *et al.*, 2010 ▸).

‡ Average of in-crystal values (Domene *et al.*, 1999 ▸; Holka *et al.*, 2014 ▸).

§ Average in-crystal value (Domene *et al.*, 1999 ▸).

¶ Estimated from the trend in ion/atom polarizability ratios for F, Cl and Br.

**Table 2:** Scale factors and fit statistics for CE-HF model energies with HF/3-21G monomer electron densities Mean absolute (MAD), mean (MD), root-mean-square (RMSD) and minimum and maximum deviations from benchmark energies are in kJ mol^−1^.

Scale factors	*k*_ele_	*k*_pol_	*k*_dis_	*k*_rep_	
	1.019 (0.882)†	0.651 (0.593)	0.901 (0.852)	0.811 (0.681)	
					
Correlation matrix	*k*_ele_	*k*_pol_	*k*_dis_	*k*_rep_	
*k*_ele_	1.000				
*k*_pol_	0.508	1.000			
*k*_dis_	0.039	0.065	1.000		
*k*_rep_	−0.314	−0.516	−0.594	1.000	
					
Fitting statistics	*N*	MAD	MD	RMSD	Min, max deviations
All pairs	1725	4.7	0.6	8.4	−44.5, 93.5
Neutral pairs	232	3.4 (2.1)†	−0.9 (0.2)	6.2 (4.0)	−42.0, 20.7
Organic salts	751	6.3	1.6	10.9	−44.5, 93.5
Metal–organics	583	3.8	0.3	6.1	−28.6, 23.9
Open shell	159	2.5	−0.8	4.7	−24.7, 11.2

† Previous values reported by Turner *et al.* (2014 ▸) are in parentheses.

**Table 3:** Scale factors and fit statistics for CE-B3LYP model energies with B3LYP/6-31G(d,p) monomer electron densities Mean absolute (MAD), mean (MD), root-mean-square (RMSD) and minimum and maximum deviations from benchmark energies are in kJ mol^−1^.

Scale factors	*k*_ele_	*k*_pol_	*k*_dis_	*k*_rep_	
	1.057 (1.063)†	0.740 (0.756)	0.871 (0.843)	0.618 (0.595)	
					
Correlation matrix	*k*_ele_	*k*_pol_	*k*_dis_	*k*_rep_	
*k*_ele_	1.000				
*k*_pol_	0.499	1.000			
*k*_dis_	0.036	0.044	1.000		
*k*_rep_	−0.344	−0.537	−0.555	1.000	
					
Fitting statistics	*N*	MAD	MD	RMSD	Min, max deviations
All pairs	1794	2.4	0.1	5.2	−69.5, 36.7
Neutral pairs	232	1.2 (1.2)†	0.3 (0.1)	2.2 (2.0)	−5.1, 12.2
Organic salts	751	4.1	0.3	7.5	−69.5, 36.7
Metal–organics	583	1.0	0.0	1.8	−14.7, 10.3
Open shell	228	1.6	−0.1	3.4	−25.0, 6.9

† Previous values reported by Turner *et al.* (2014 ▸) are in parentheses.

**Table 4:** CE-B3LYP and PIXEL estimates of energy components and total energies for the closest intermolecular interactions in Cr(CO)_6_ All energies are in kJ mol^−1^ and PIXEL results are from Maloney *et al.* (2015 ▸).

Cr⋯Cr distance (Å)		*E*_ele_	*E*_pol_	*E*_dis_	*E*_rep_	*E*_tot_
6.203†	CE-B3LYP	−5.8	−0.4	−13.9	8.3	−11.7
6.208	CE-B3LYP	−3.2	−0.4	−12.9	5.9	−10.4
	***PIXEL***	***−2.6***	***−1.2***	***−16.9***	***8.9***	***−11.9***
6.244	CE-B3LYP	−6.0	−0.4	−13.9	8.8	−11.5
	***PIXEL***	***−5.8***	***−1.8***	***−18.7***	***13.0***	***−13.3***
6.882	CE-B3LYP	−4.4	−0.3	−8.4	5.0	−8.0
	***PIXEL***	***−4.3***	***−1.1***	***−11.2***	***7.4***	***−9.1***

† PIXEL results not reported for this pair by Maloney *et al.* (2015 ▸).

**Table 5:** CE-B3LYP interaction energies and *J*_AB_ exchange couplings for the nine radical pairs in *p*-(methylthio)phenyl nitro­nyl nitroxide considered by Deumal *et al.* (2004 ▸). CE-B3LYP energies and exchange couplings were computed for three crystal geometries at 10 (CSD refcode YUJNEW12), 114 (YUJNEW11) and 298 K [YUJNEW, with H atoms added using *Mercury* (Macrae *et al.*, 2008 ▸)] Centroid distances in Å, interaction energies in kJ mol^−1^ and exchange couplings in cm^−1^. Estimated uncertainties are ∼1 kJ mol^−1^ in *E*_tot_ and ∼0.02 cm^−1^ for *J*_AB_.

Radical pair	Centroid distances (10, 114, 298 K)	*E*_tot_ (10 K)	*E*_tot_ (114 K)	*E*_tot_ (298 K)	*J*_AB_ (10 K)	*J*_AB_ (114 K)	*J*_AB_ (298 K)
d1	4.62, 4.67, 4.74	−59	−58	−54	+0.22	+0.11	+0.24
d2	7.86, 7.88, 7.92	−16	−16	−16	+0.07	+0.07	+0.09
d3	8.60, 8.63, 8.52	−20	−20	−21	−0.02	−0.02	−0.11
d4	9.96, 10.00, 10.14	−14	−14	−13	†	†	‡
d5	10.85, 10.95, 11.23	−6	−5	−6	†	†	‡
d6	11.61, 11.67, 11.84	−8	−8	−3	+0.08	+0.07	+0.02
d7	10.62, 10.65, 10.65	−1	−1	−1	†	†	‡
d8	11.19, 11.22, 11.28	−6	−6	−6	†	†	‡
d9	13.49, 13.56, 13.53	−8	−8	−8	†	†	‡

† |*J*_AB_| ≤ 10^−2^ according to Deumal *et al.* (2004 ▸).

‡ Not reported by Deumal *et al.* (2004 ▸); presumably small.

## Footnotes

1 We note that because the PIXEL and CE-B3LYP electrostatic *and* total energy values are in excellent agreement, then on average the sum of the other three terms for the two schemes differ by a scale factor.

## References

[bb1] Acree, W. & Chickos, J. S. (2016). *J. Phys. Chem. Ref. Data*, **45**, 033101.

[bb2] Acree, W. & Chickos, J. S. (2017). *J. Phys. Chem. Ref. Data*, **46**, 013104.

[bb3] Allen, F. H., Watson, D. G., Brammer, L., Orpen, A. G. & Taylor, R. (2004). *International Tables for Crystallography*, 3rd ed., edited by E. Prince, pp. 790–811. Berlin: Springer Verlag.

[bb4] Azuri, I., Meirzadeh, E., Ehre, D., Cohen, S. R., Rappe, A. M., Lahav, M., Lubomirsky, I. & Kronik, L. (2015). *Angew. Chem. Int. Ed.* **54**, 13566–13570. 10.1002/anie.201505813 26373817

[bb5] Bader, R. F. W. (1990). In *Atoms in Molecules - A Quantum Theory.* Oxford: Oxford University Press.

[bb6] Bogdanović, G. A. & Novaković, S. B. (2011). *CrystEngComm*, **13**, 6930–6932.

[bb7] Chickos, J. S. (2003). *Netsu Sokutei*, **39**, 116–124.

[bb8] Desiraju, G. R. (1989). In *Crystal Engineering: The Design of Organic Solids*. Amsterdam: Elsevier.

[bb9] Desiraju, G. R. (1997). *Chem. Commun.* pp. 1475–1482.

[bb10] Destro, R., Roversi, P., Barzaghi, M. & Marsh, R. E. (2000). *J. Phys. Chem. A*, **104**, 1047–1054.

[bb11] Deumal, M., Bearpark, M. J., Robb, M. A., Pontillon, Y. & Novoa, J. J. (2004). *Chem. Eur. J.* **10**, 6422–6432. 10.1002/chem.200400493 15532054

[bb12] Dey, D., Bhandary, S., Thomas, S. P., Spackman, M. A. & Chopra, D. (2016*a*). *Phys. Chem. Chem. Phys.* **18**, 31811–31820. 10.1039/c6cp05917a 27841399

[bb13] Dey, D., Thomas, S. P., Spackman, M. A. & Chopra, D. (2016*b*). *Chem. Commun.* **52**, 2141–2144. 10.1039/c5cc09741j 26693707

[bb14] Domene, C., Fowler, P. W., Madden, P. A., Wilson, M. & Wheatley, R. J. (1999). *Chem. Phys. Lett.* **314**, 158–167.

[bb15] Dunitz, J. D. (2015). *IUCrJ*, **2**, 157–158. 10.1107/S2052252515002006 PMC4392407 25866649

[bb16] Dunitz, J. D. & Gavezzotti, A. (2012). *J. Phys. Chem. B*, **116**, 6740–6750. 10.1021/jp212094d 22360776

[bb17] Edwards, A. J., Mackenzie, C. F., Spackman, P. R., Jayatilaka, D. & Spackman, M. A. (2017). *Faraday Discuss.* doi:10.1039/C7FD00072C. 10.1039/c7fd00072c 28721418

[bb18] Eikeland, E., Spackman, M. A. & Iversen, B. B. (2016*a*). *Cryst. Growth Des.* **16**, 6858–6866.

[bb19] Eikeland, E., Thomsen, M. K., Madsen, S. R., Overgaard, J., Spackman, M. A. & Iversen, B. B. (2016*b*). *Chem. Eur. J.* **22**, 4061–4069. 10.1002/chem.201504908 26879515

[bb20] Espinosa, E., Molins, E. & Lecomte, C. (1998). *Chem. Phys. Lett.* **285**, 170–173.

[bb21] Frisch, M. J., *et al.* (2009). *GAUSSIAN09*. Gaussian Inc., Wallingford, CT, USA. http://www.gaussian.com.

[bb22] Gavezzotti, A. (2002). *J. Phys. Chem. B*, **106**, 4145–4154.

[bb23] Gavezzotti, A. (2003). *J. Phys. Chem. B*, **107**, 2344–2353.

[bb24] Gavezzotti, A. (2005). *Z. Kristallogr.* **220**, 499–510.

[bb25] Gavezzotti, A. (2008). *Mol. Phys.* **106**, 1473–1485.

[bb26] Gavezzotti, A. & Filippini, G. (1997). *Theoretical Aspects and Computer Modeling of the Molecular Solid State*, edited by A. Gavezzotti, pp. 61–97. Chichester: Wiley.

[bb27] Gelbrich, T., Braun, D. E. & Griesser, U. J. (2016). *Chem. Cent. J.* **10**, 8. 10.1186/s13065-016-0152-5 PMC4763432 26909105

[bb28] Grimme, S. (2006). *J. Comput. Chem.* **27**, 1787–1799. 10.1002/jcc.20495 16955487

[bb29] Hayes, I. C. & Stone, A. J. (1984). *Mol. Phys.* **53**, 83–105.

[bb100] Groom, C. R., Bruno, I. J., Lightfoot, M. P. & Ward, S. C. (2016). *Acta Cryst.* B**72**, 171–179. 10.1107/S2052520616003954 PMC4822653 27048719

[bb30] Holka, F., Urban, M., Neogrády, P. & Paldus, J. (2014). *J. Chem. Phys.* **141**, 214303. 10.1063/1.4902353 25481140

[bb31] Jeziorski, B., Moszynski, R. & Szalewicz, K. (1994). *Chem. Rev.* **94**, 1887–1930.

[bb32] Johnson, E. R., Keinan, S., Mori-Sánchez, P., Contreras-García, J., Cohen, A. J. & Yang, W. T. (2010). *J. Am. Chem. Soc.* **132**, 6498–6506. 10.1021/ja100936w PMC2864795 20394428

[bb33] Kitaura, K. & Morokuma, K. (1976). *Int. J. Quantum Chem.* **10**, 325–340.

[bb34] Kvick, Å., Canning, W. M., Koetzle, T. F. & Williams, G. J. B. (1980). *Acta Cryst.* B**36**, 115–120.

[bb35] Lai, F., Du, J. J., Williams, P. A., Váradi, L., Baker, D., Groundwater, P. W., Overgaard, J., Platts, J. A. & Hibbs, D. E. (2016). *Phys. Chem. Chem. Phys.* **18**, 28802–28818. 10.1039/c6cp02690g 27722530

[bb36] Landeros-Rivera, B., Moreno-Esparza, R. & Hernández-Trujillo, J. (2016). *RSC Adv.* **6**, 77301–77309.

[bb37] Langan, P., Mason, S. A., Myles, D. & Schoenborn, B. P. (2002). *Acta Cryst.* B**58**, 728–733. 10.1107/s0108768102004263 12149564

[bb38] Lecomte, C., Espinosa, E. & Matta, C. F. (2015). *IUCrJ*, **2**, 161–163. 10.1107/S2052252515002067 PMC4392409 25866651

[bb39] Macrae, C. F., Bruno, I. J., Chisholm, J. A., Edgington, P. R., McCabe, P., Pidcock, E., Rodriguez-Monge, L., Taylor, R., van de Streek, J. & Wood, P. A. (2008). *J. Appl. Cryst.* **41**, 466–470.

[bb40] Makal, A. M., Plażuk, D., Zakrzewski, J., Misterkiewicz, B. & Woźniak, K. (2010). *Inorg. Chem.* **49**, 4046–4059. 10.1021/ic9019958 20369823

[bb41] Maloney, A. G. P., Wood, P. A. & Parsons, S. (2015). *CrystEngComm*, **17**, 9300–9310.

[bb42] Maschio, L., Civalleri, B., Ugliengo, P. & Gavezzotti, A. (2011). *J. Phys. Chem. A*, **115**, 11179–11186. 10.1021/jp203132k 21894880

[bb43] Mitroy, J., Safronova, M. S. & Clark, C. W. (2010). *J. Phys. B At. Mol. Opt. Phys.* **43**, 202001.

[bb44] Moggach, S. A., Marshall, W. G., Rogers, D. M. & Parsons, S. (2015). *CrystEngComm*, **17**, 5315–5328.

[bb45] Nangia, A. & Desiraju, G. R. (1998). *Design of Organic Solids*, edited by E. Weber, pp. 57–95. Berlin: Springer-Verlag.

[bb46] Nyman, J. & Day, G. M. (2015). *CrystEngComm*, **17**, 5154–5165.

[bb47] Otero-de-la-Roza, A. & Johnson, E. J. (2012). *J. Chem. Phys.* **137**, 054103. 10.1063/1.4738961 22894328

[bb48] Perlovich, G. L., Hansen, L. K. & Bauer-Brandl, A. (2001). *J. Therm. Anal. Calorim.* **66**, 699–715.

[bb49] Pillet, S., Souhassou, M., Pontillon, Y., Caneschi, Y., Gatteschi, D. & Lecomte, C. (2001). *New J. Chem.* **25**, 131–143.

[bb50] Pontillon, Y., Caneschi, A., Gatteschi, D., Grand, A., Ressouche, E., Sessoli, R. & Schweizer, J. (1999). *Chem. Eur. J.* **5**, 3616–3624.

[bb51] Pyziak, M., Pyziak, J., Hoffmann, M. & Kubicki, M. (2015). *Cryst. Growth Des.* **15**, 5223–5232.

[bb52] Shi, M. W., Thomas, S. P., Koutsantonis, G. A. & Spackman, M. A. (2015). *Cryst. Growth Des.* **15**, 5892–5900.

[bb53] Spackman, M. A. (2013). *Phys. Scr.* **87**, 048103.

[bb54] Spackman, M. A. (2015). *Cryst. Growth Des.* **15**, 5624–5628.

[bb55] Spackman, M. A. & Jayatilaka, D. (2009). *CrystEngComm*, **11**, 19–32.

[bb56] Su, P. & Li, H. (2009). *J. Chem. Phys.* **131**, 014102. 10.1063/1.3159673 19586091

[bb57] Thakkar, A. J. & Lupinetti, C. (2006). *Atoms, Molecules and Clusters in Electric Fields*, edited by G. Maroulis, pp. 505–529. Singapore: World Scientific.

[bb58] Thakur, T. S., Dubey, R. & Desiraju, G. R. (2015). *IUCrJ*, **2**, 159–160. 10.1107/S205225251500189X PMC4392408 25866650

[bb59] Turner, M. J., Grabowsky, S., Jayatilaka, D. & Spackman, M. A. (2014). *J. Phys. Chem. Lett.* **5**, 4249–4255. 10.1021/jz502271c 26273970

[bb60] Turner, M. J., McKinnon, J. J., Jayatilaka, D. & Spackman, M. A. (2011). *CrystEngComm*, **13**, 1804–1813.

[bb61] Turner, M. J., McKinnon, J. J., Wolff, S. K., Grimwood, D. J., Spackman, P. R., Jayatilaka, D. & Spackman, M. A. (2017). *CrystalExplorer17*. University of Western Australia. http://hirshfeldsurface.net

[bb62] Turner, M. J., Thomas, S. P., Shi, M. W., Jayatilaka, D. & Spackman, M. A. (2015). *Chem. Commun.* **51**, 3735–3738. 10.1039/c4cc09074h 25525647

[bb63] Wiechert, D. & Mootz, D. (1999). *Angew. Chem. Int. Ed.* **38**, 1974–1976. 10.1002/(SICI)1521-3773(19990712)38:13/14<1974::AID-ANIE1974>3.0.CO;2-F 34182670

[bb64] Zhurov, V. V. & Pinkerton, A. A. (2015). *J. Phys. Chem. A*, **119**, 13092–13100. 10.1021/acs.jpca.5b10027 26618800

[bb65] Ziegler, T. & Rauk, A. (1979). *Inorg. Chem.* **18**, 1755–1759.

