# Supplementary material for: CrystalExplorer model energies and energy frameworks: extension to metal coordination compounds, organic salts, solvates and open-shell systems
Source: IUCrJ. 2017 Jul 4;4(Pt 5):575–87. doi: 10.1107/S205225251700848X (PMC5600021; doi:10.1107/S205225251700848X)
Supplement: Supplementary file 1 [file m-04-00575-sup1.pdf]

IUCrJ

Volume 4 (2017)

Supporting information for article:

CrystalExplorer model energies and energy frameworks: extension to metal coordination compounds, organic salts, solvates and open-shell systems

Campbell F. Mackenzie, Peter R. Spackman, Dylan Jayatilaka and Mark A. Spackman

Table S1

Table S2 Crystal structure details for 37 “neutral pairs”.

	**Name**	**Diagram**	**Reference**
15318-<br>ICSD-I2	iodine	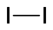	F. van Bolhuis, P.B. Koster, T. Migchelsen, *Acta Cryst.* (1967) **23**, 90.
200455-<br>ICSD-S8	sulfur	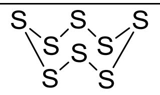	P. Coppens, Y.W. Yang, R.H. Blessing,W.F. Cooper,F.K. Larsen, *J. Am. Chem. Society* (1977) **99**, 760.
201142-<br>ICSD-N2O4	dinitrogen tetroxide	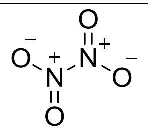	A. Kvick, R.K. McMullan, M.D Newton, *J. Chem. Phys.* (1982) **76**, 3754.
201693-<br>ICSD-Br2	bromine	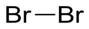	B.M. Powell, K.M. Heal, B.H. Torrie, *Mol. Phys.* (1984) **53**, 929.
201698-<br>ICSD-Cl2	chlorine	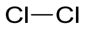	B.M. Powell, K.M. Heal, B.H. Torrie, *Mol. Phys.* (1984) **53**, 929.
260950-<br>ICSD-XeF2	xenon difluoride	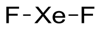	H.S.A. Elliot, J.F. Lehmann, H.P.A. Mercier, H.D.B. Jenkins, G.J. Schrobilgen, *Inorg. Chem.* (2010) **49**, 8504.

30501-ICSD-disiloxane	disiloxane	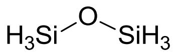	M.J. Barrow,E.A.V. Ebsworth, M.M. Harding, *Acta Cryst. B* (1979) **35**, 2093.
ACETYL02	acetylene	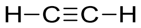	R.K.McMullan, A.Kvick, P.Popelier, *Acta Crystallogr.,Sect.B:Struct.Sci.* (1992), **48**, 726.
ACOKEI	4-bromo-2,3,5,6-fluorobenzonitrile	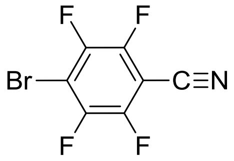	A.D.Bond, J.Griffiths, J.M.Rawson, J.Hulliger, *Chem.Commun.* (2001), 2488.
AEPHOS02	phosphorylethanolamine	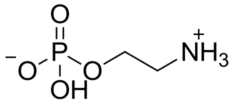	H.-P.Weber, R.K.McMullan, S.Swaminathan, B.M.Craven, *Acta Crystallogr.,Sect.B:Struct.Sci.* (1984), **40**, 506.
ARSACP02	phenylarsonic acid	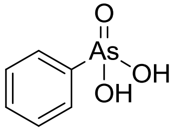	N.C.Lloyd, H.W.Morgan, B.K.Nicholson, R.S.Ronimus, *J.Organomet.Chem.* (2008), **693**, 2443.
BENZEN01	benzene	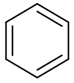	G.E.Bacon, N.A.Curry, S.A.Wilson, *Proc.R.Soc.London,Ser.A* (1964), **279**, 98.
DCLBEN03 -gamma	p-dichlorobenzene	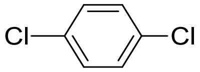	G.L.Wheeler, S.D.Colson, *Acta Crystallogr.,Sect.B:Struct.Crystallogr.Cryst.Chem.* (1975), **31**, 911.
DCLBEN06 -beta	p-dichlorobenzene	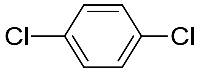	G.L.Wheeler, S.D.Colson, *J.Chem.Phys.* (1976), **65**, 1227.

DCLBEN07 -alpha	p-dichlorobenzene	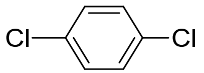	G.L.Wheeler, S.D.Colson, *J.Chem.Phys.* (1976), **65**, 1227.
EBIHIH	1,4-dicyanobutane 1,2-di-iodo-1,1,2,2-tetrafluoroethane	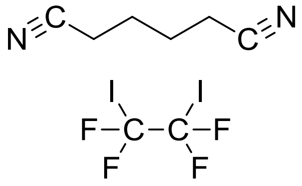	P.Metrangolo, T.Pilati, G.Resnati, A.Stevenazzi, *Chem.Commun.* (2004), 1492.
ETHANE01	ethane	H_3C-CH_3	G.J.H.van Nes, A.Vos, *Acta Crystallogr.,Sect.B:Struct.Crystallogr.Cryst.Chem.* (1978), **34**, 1947.
ETHLEN10	ethylene	$H_2C=CH_2$	G.J.H.van Nes, A.Vos, *Acta Crystallogr.,Sect.B:Struct.Crystallogr.Cryst.Chem.* (1979), **35**, 2593.
FORMAC01	formic acid	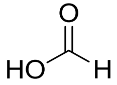	I.Nahringbauer, *Acta Crystallogr.,Sect.B:Struct.Crystallogr.Cryst.Chem.* (1978), **34**, 315.
FORMAM02	formamide	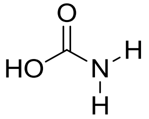	E.D.Stevens, *Acta Crystallogr.,Sect.B:Struct.Crystallogr.Cryst.Chem.* (1978), **34**, 544.
IMAZOL13	imidazole	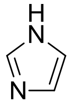	B.M.Craven, R.K.McMullan, J.D.Bell, H.C.Freeman, *Acta Crystallogr.,Sect.B:Struct.Crystallogr.Cryst.Chem.* (1977), **33**, 2585.
JAGREP	orthoboric acid	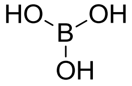	R.R.Shuvalov, P.C.Burns, *Acta Crystallogr.,Sect.C:Cryst.Struct.Commun.* (2003), **59**, i47.

JOYHUA	4H-selenopyran-4-one	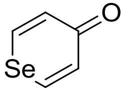	M.R.Detty, H.R.Luss, *Organometallics* (1992), **11**, 2157.
JUPPAL	5-amino-2-mesyl-2,3-dihydro-1,2,4-oxadiazol-3-one	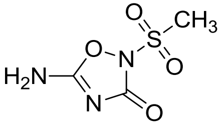	Do Young Ra, Nam Sook Cho, Sung Kwon Kang, Eun Suk Choi, Il Hwan Suh, *J.Chem.Soc.,Perkin Trans.2* (1999), 81.
LALNIN03	L-alanine	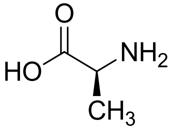	R.Destro, R.E.Marsh, R.Bianchi, *J.Phys.Chem.* (1988), **92**, 966.
MEADEN05	9-methyladenine	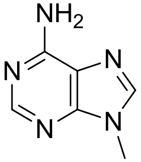	K.N.Jarzembska, A.M.Goral, R.Gajda, P.M.Dominiak, *Cryst.Growth Des.* (2013), **13**, 239.
MTHMAD11	9-methyladenine-1-methylthymine complex	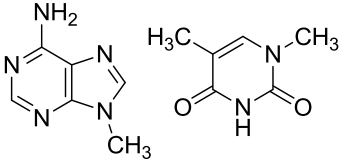	M.N.Frey, T.F.Koetzle, M.S.Lehmann, W.C.Hamilton, *J.Chem.Phys.* (1973), **59**, 915.
NAPHQU	1,4-naphthoquinone	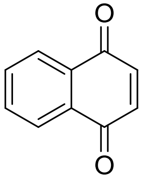	J.Gaultier, C.Hauw, *Acta Crystallogr.* (1965), **18**, 179.

NTRGUA03	2-nitroguanidine	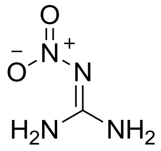	R.K.Murmann, R.Glaser, C.L.Barnes, *J.Chem.Cryst.* (2005), **35**, 317.
PVVAWA0<br>1	1,3,5-trifluorobenzene	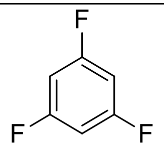	V.R.Thalladi, H.-C.Weiss, D.Blaser, R.Boese, A.Nangia, G.R.Desiraju, *J.Am.Chem.Soc.* (1998), **120**, 8702.
PYRIDO04	2-pyridone	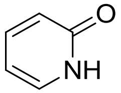	U.Ohms, H.Guth, E.Hellner, H.Dannohl, A.Schweig, *Z.Kristallogr.* (1984), **169**, 185.
QEWXIA01	2,3-dichlorobenzaldehyde	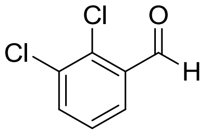	K.A.Solanko, A.D.Bondy, *Acta Crystallogr.,Sect.B:Struct.Sci.* (2011), **67**, 437.
SUCACB09	1,4-butanedioic acid	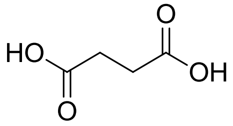	V.R.Thalladi, M.Nusse, R.Boese, *J.Am.Chem.Soc.* (2000), **122**, 9227.
URACIL	uracil	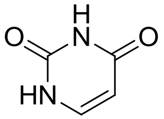	R.F.Stewart, L.H.Jensen, *Acta Crystallogr.* (1967), **23**, 1102.
UREAXX12	urea	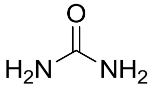	S.Swaminathan, B.M.Craven, R.K.McMullan, *Acta Crystallogr.,Sect.B:Struct.Sci.* (1984), **40**, 300.

VAMZIT01	pentacyclo[4.4.0.0^{2,5}.0^{3,8}.0^{4,7}]dec-9-ene (basketene)	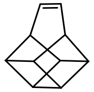	S.P.Verevkin, M.Kummerlin, E.Hickl, H.-D.Beckhaus, C.Ruchardt, S.I.Kozhushkov, R.Haag, R.Boese, J.Benet-Bucholz, K.Nordhoff, A.de Meijere, *Eur.J.Org.Chem.* (2002), 2280.
VITRUL	3,5-dichloro-1H-1,2,4-triazole	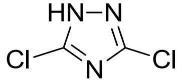	G.L.Starova, O.V.Frank-Kamenetskaya, V.V.Makarskii, *Kristallografiya(Russ.)(Crystallogr.Rep.)* (1990), **35**, 769

Table S3 Crystal structure details for 44 organic salts, including solvates.

ANLINC01	anilinium chloride	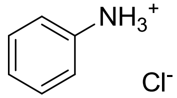	E.Lopez-Dupla, P.G.Jones, F.Vancea, *Z.Naturforsch.,B:Chem.Sci.* (2003).
BEPTUN	morpholin-4-ium acetate	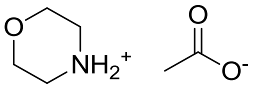	S.P.Kelley, A.Narita, J.D.Holbrey, K.D.Green, W.M.Reichert, R.D.Rogers, *Cryst.Growth Des.* (2013), **13**, 965.
BOGCAC	benzylammonium chloride	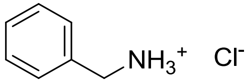	M.Topper, M.L.McLaughlin, F.R.Fronczek, *CSD Communication(Private Communication)*
COXYIY	3-(2-hydroxy(diphenyl)acetoxy)-1,1-dimethylpiperidinium 3-oxo-3H-1,2-benzothiazol-2-ide 1,1-dioxide<br><br>(mepenzolate saccharinate)	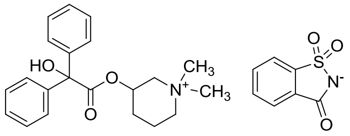	P.M.Dean, J.Turanjanin, M.Yoshizawa-Fujita, D.R.MacFarlane, J.L.Scott, *Cryst.Growth Des.* (2009), **9**, 1137.
CUZHEK	calcium α-ethylmalonate	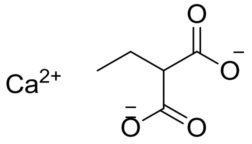	A.Zell, H.Einspahr, C.E.Bugg, *Biochemistry* (1985), **24**, 533.
DEZYEO	1-phenylethanaminium 2-methylbenzoate	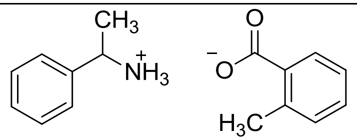	T.Sasaki, I.Hisaki, T.Miyano, N.Tohnai, K.Morimoto, H.Sato, S.Tsuzuki, M.Miyata, *Nat.Commun.* (2013), **4**, 1787.

DUBWAZ	tetramethylammonium 2-(acetylamino)propanoate	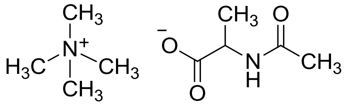	T.Gelbrich, E.Shepherd, M.B.Hursthouse, J.D.Kilburn, *University of Southampton, Crystal Structure Report Archive* (2000), 405.
DUFBIP	potassium o-sulfonatobenzoic acid	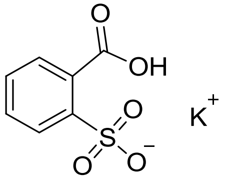	T.B.Teplova, T.N.Turskaya, T.A.Shibanova, Yu.V.Nekrasov, G.S.Belikova, *Kristallografiya(Russ.)(Crystallogr.Rep.)* (1986), **31**, 187.
DUVCAZ	4-ethylanilinium 2-carboxyacetate	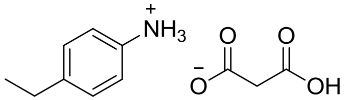	De-Hong Wu, Qi-Qi Wu, *Acta Crystallogr.,Sect.E:Struct.Rep.Online* (2010), **66**, o2160.
FENYEC	pyrrolidinium p-hydroxybenzoate	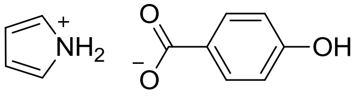	Y.Moritani, N.Sasahara, S.Kashino, M.Haisa, *Acta Crystallogr.,Sect.C:Cryst.Struct.Commun.* (1987), **43**, 154.
HEZXAM	2-hydroxyethanaminium (S)-hydroxy(phenyl)acetate<br><br>(ethanolammonium mandelate)	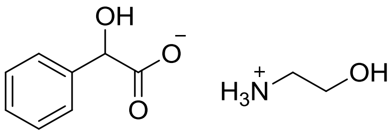	N.Wermester, E.Aubin, M.Pauchet, S.Coste, G.Coquerel, *Tetrahedron:Asymm.* (2007), **18**, 821.
HISREG	(R)-(+)-1 phenylethylammonium chloroacetate	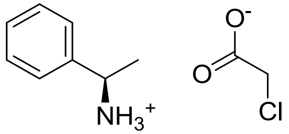	S.Houllemare-Druot, G.Coquerel, *J.Chem.Soc.,Perkin Trans.2* (1998), 2211.
KUDFIA	2-(dimethylamino)ethanaminium benzoate	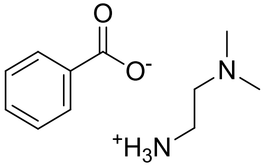	Dittrich, Dennis, Wolper, Christoph, Schulz, Stephan, *CSD Communication(Private Communication)*

LARASC20	L-arginine L-ascorbate	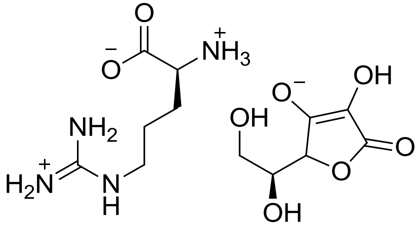	V.Sudhakar, M.Vijayan, *Acta Crystallogr.,Sect.B:Struct.Crystallogr.Cryst.Chem.* (1980), **36**, 120.
LIHMAM	lithium hydrogen (+)-1-malate	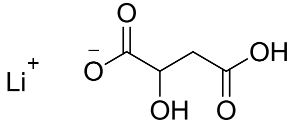	W.Van Havere, A.T.H.Lenstra, *Acta Crystallogr.,Sect.B:Struct.Crystallogr.Cryst.Chem.* (1980), **36**, 1483.
MATPHB	tetramethylammonium tetraphenylborate	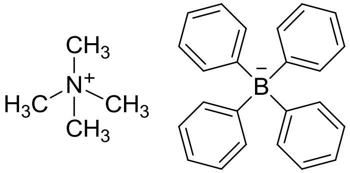	K.Hoffmann, E.Weiss, *J.Organomet.Chem.* (1974), **67**, 221.
MEDHOU01	2-(4-hydroxyphenyl)ethanaminium hydroxy(phenyl)acetate	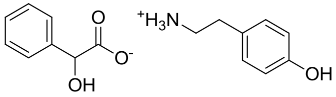	N.E.B.Briggs, A.R.Kennedy, C.A.Morrison, *Acta Crystallogr.,Sect.B:Struct.Sci.* (2012), **68**, 453.
MIBTOH	n-butylammonium triphenylacetate	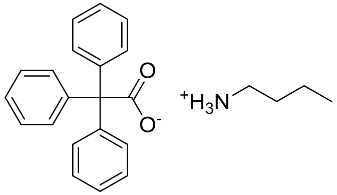	T.Yuge, N.Tohnai, T.Fukuda, I.Hisaki, M.Miyata, *Chem.-Eur.J.* (2007), **13**, 4163.

NAASCB	sodium ascorbate	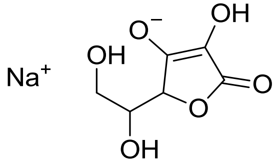	J.Hvoslef, *Acta Crystallogr.,Sect.B:Struct.Crystallogr.Cryst.Chem.* (1969), **25**, 2214.
NAHCIT	sodium dihydrogen citrate	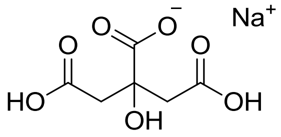	J.P.Glusker, D.van der Helm, W.E.Love, M.L.Dornberg, J.A.Minkin, C.K.Johnson, A.L.Patterson, *Acta Crystallogr.* (1965), **19**, 561.
PAMBZA03	4-carboxyanilinium chloride	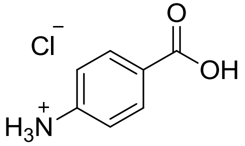	Wen Wu, Jiming Xie, Dongpo Xie, *Zh.Neorg.Khim.(Russ.)(Russ.J.Inorg.Chem.)* (2010), **55**, 384.
PAXNIN	4-hydroxyanilinium carboxyacetate	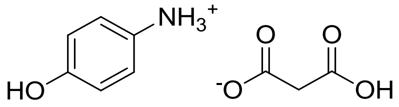	Ying-Chun Wang, *Acta Crystallogr.,Sect.E:Struct.Rep.Online* (2012), **68**, o1937.
RIRMUA	potassium phthalohydroxamate	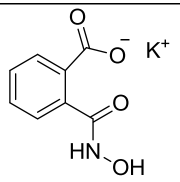	Z.Ch.Kadyrova, N.K.Makhmudova, Kh.T.Sharipov, N.A.Parpiev, *Uzb.Khim.Zh.(Russ.)(Uzbek Journal on Chemistry)* (1996), 11-5.
SIYSEA	umifenovir hydrogen maleate	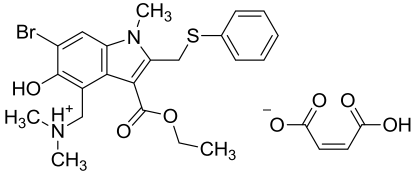	L.Orola, I.Sarcevica, A.Kons, A.Actins, M.V.Veidis, *J.Mol.Struct.* (2014), **1056**, 63.

TRPPAC	tryptammonium phenylacetate	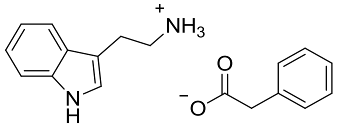	M.Inoue, T.Sakaki, T.Fujiwara, K.Tomita, *Bull.Chem.Soc.Jpn.* (1978), **51**, 1123.
TUYKAB	(1R,2S)-norephedrine 3,5-dimethoxybenzoate	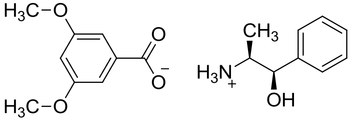	Chunji Li, J.Cho, K.Yamada, D.Hashizume, F.Araoka, H.Takezoe, T.Aida, Y.Ishida, *Nat.Commun.* (2015), **6**, 8418.
YABFOY	cyclohexylammonium acetate	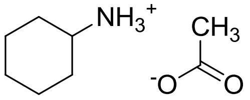	J.A.Odendal, J.C.Bruce, K.R.Koch, D.A.Haynes, *CrystEngComm* (2010), **12**, 2398,
ZOWWEP	ammonium phenoxyacetate	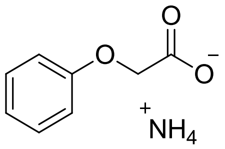	G.Smith, *Acta Crystallogr.,Sect.E:Struct.Rep.Online* (2014), **70**, 528,
FOVCAV	2-methyl-5-nitroanilinium trichloroacetate monohydrate	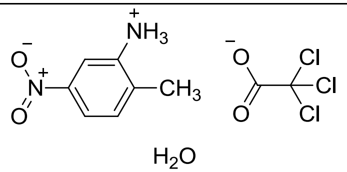	J.Janczak, G.J.Perpetuo, *Acta Crystallogr.,Sect.C:Cryst.Struct.Commun.* (2009), **65**, o339.
GELSUM	(2S,1'R)-2-(1'-phenylethylcarbamoyl)pyrrolidinium trifluoroacetate benzene solvate	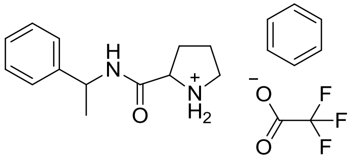	S.S.Chimni, D.Mahajan, *Tetrahedron:Asymm.* (2006), **17**, 2108.
HODPUM	4-benzylpiperazin-1-ium chloride chloroform solvate	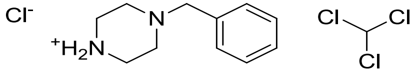	M.G.Nema, R.A.Varga, C.Silvestru, H.J.Breunig, *Acta Crystallogr.,Sect.E:Struct.Rep.Online* (2008), **64**, o1585.

HORVAL	di-isopropylammonium chloride chloroform solvate	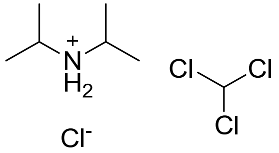	M.Nieger, E.Niecke, A.Loew, *CSD Communication(Private Communication)* (1999)
HURMOX	2-hydroxy-1,2-diphenylethanaminium 2-thienylacetate butan-1-ol solvate	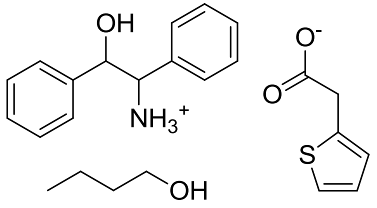	N.Shiota, T.Kinuta, T.Sato, R.Kuroda, Y.Matsubara, Y.Imai, *Tetrahedron* (2010), **66**, 5589.
IDOPAT	N-((S)-1-(5-methyl-2-hydroxyphenyl)ethyl)-N-((R)-2-hydroxy-1-phenylethyl)amine chloride ethyl acetate solvate	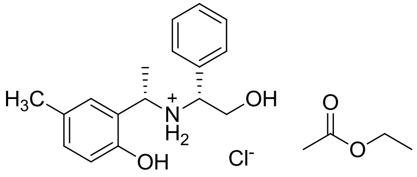	Guang-You Zhang, Xiang-Bo Wang, Jin-Yan Zhao, Wan-Hui Wang, Xiao-Feng Yang, *Acta Crystallogr.,Sect.E:Struct.Rep.Online* (2006), **62**, o3035.
IRURAO	diisopropylammonium triphenylacetate benzene solvate	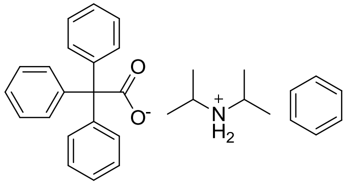	K.Sada, T.Watanabe, J.Miyamoto, T.Fukuda, N.Tohnai, M.Miyata, T.Kitayama, K.Maehara, K.Ute, *Chem.Lett.* (2004), **33**, 160.
ISIHIB	tetramethylammonium benzoate monohydrate	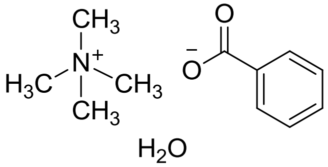	H.-Stoeckli-Evans, G.Aromi, *CSD Communication(Private Communication)* (2004)

JAMMIT	(3,4-dihydroxyphenylacyl)trimethylammonium chloride methanol solvate	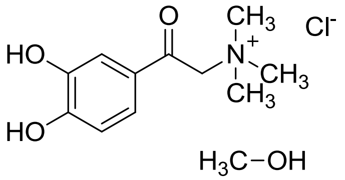	R.B.Barlow, O.Johnson, J.A.K.Howard, D.C.Walton, G.Koellner, *Acta Crystallogr.,Sect.B:Struct.Sci.* (1989), **45**, 396.
KOLDUL	(1R,2R)-1,2-diphenylethylenediammonium N-phenyliminodiacetate ethanol clathrate	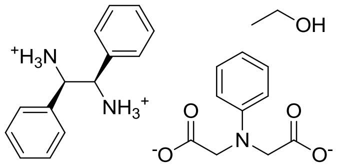	Y.Imai, K.Murata, T.Sato, R.Kurodao, Y.Matsubara, *Org.Lett.* (2008), **10**, 3821.
MUTWUT	sodium 1-(α-L-threofuranosyl)thymine acetate	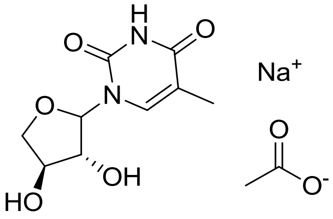	K.-U.Schoning, P.Scholz, Xiaolin Wu, S.Guntha, G.Delgado, R.Krishnamurthy, A.Eschenmoser, *Helv.Chim.Acta* (2002), **85**, 4111.
ROJJEG	N,N-dimethyl-3-phthalimidopropylammonium acetate hemihydrate	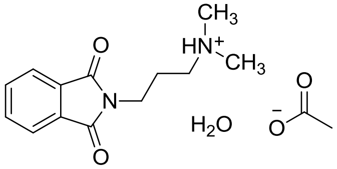	T.Borowiak, I.Wolska, P.Jensz, I.Kowalczyk, B.Brycki, A.Sztul, *J.Mol.Struct.* (2008), **891**, 205.
SIMRAI	isopropylammonium (isopropylamino)oxoacetate monohydrate	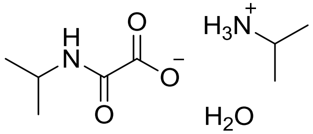	X.Sheng, C.E.Strasser, H.G.Raubenheimer, R.C.Luckay, *Acta Crystallogr.,Sect.E:Struct.Rep.Online* (2007), **63**, o4361.

UGULUF	benzylammonium (4-chlorophenoxy)acetate monohydrate	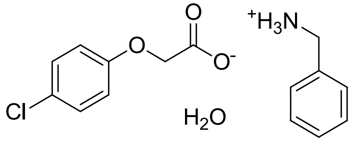	Shouwen Jin, Zhanghui Lin, Daqi Wang, Guqing Chen, Zuoyi Ji, Tiansong Huang, Ying Zhou, *J.Chem.Cryst.* (2015), **45**, 159.
VEMJII	2-hydroxy-3-phenoxypropan-1-aminium hydroxy(phenyl)acetate monohydrate	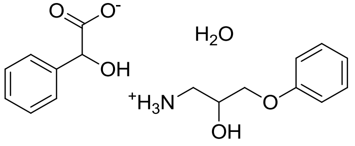	Pei Wang, En Zhang, Jian-Feng Niu, Qing-Hua Ren, Peng Zhao, Hong-Min Liu, *Tetrahedron:Asymm.* (2012), **23**, 1046,
YEFVAH	(2S)-methyl 2-ammonio-3-(4-hydroxyphenyl)propionate chloride methanol solvate<br><br>(L-tyrosine methyl ester hydrochloride methanol solvate)	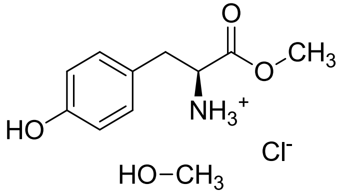	I.Bryndal, M.Jaremko, L.Jaremko, T.Lis, *Acta Crystallogr.,Sect.C:Cryst.Struct.Commun.* (2006), **62**, o111

Table S4   Crystal structure details for 63 metal organics, including solvates.

ACACCS	bis(2,4-pentanedionato) chromium(II)	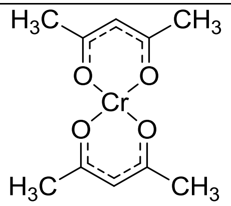	F.A.Cotton, C.E.Rice, G.W.Rice, *Inorg.Chim.Acta* (1977), **24**, 231.
ACACMN02	tris(acetylacetonato)-manganese(iii)	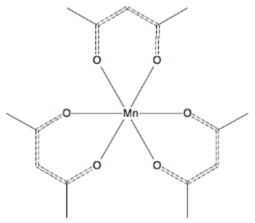	J.P.Fackler Junior, A.Avdeef, *Inorg.Chem.* (1974), **13**, 1864
ACACSC	tris(acetylacetonato)-scandium(III)	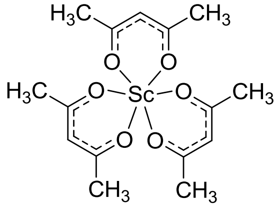	T.J. Anderson, M.A. Neuman, G.A. Melson, *Inorg. Chem.* (1973), **12**, 927.
BENDAB	(4,4'-dimethyl-2,2'-bipyridine)-diacetato-zinc(II)	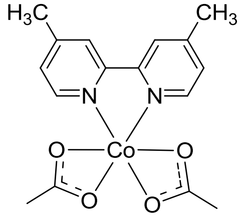	M.A.Harvey, S.A.Suarez, A.Ibanez, F.Doctorovich, R.Baggio, *Acta Crystallogr.,Sect.E:Struct.Rep.Online* (2012), **68**, m1377.

BZCRCO	(η^6-benzene)-tricarbonyl-chromium(0)	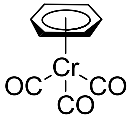	M.F.Bailey, L.F.Dahl, *Inorg.Chem.* (1965), **4**, 1314.
CCRTOL	(η^6-toluene)-tricarbonyl-chromium(0)	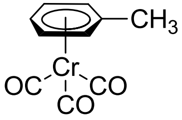	F.van Meurs, H.van Koningsveld, *J.Organomet.Chem.* (1977), **131**, 42.
CEFFOI	(2,2'-bipyridine)-dichloro-zinc(ii)	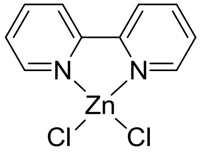	M.A.Khan, D.G.Tuck, *Acta Crystallogr.,Sect.C:Cryst.Struct.Commun.* (1984), **40**, 60.
CEHPIO01	trichloro-(η^5-cyclopentadienyl)-titanium(IV)	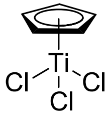	A.J.Rossini, R.W.Mills, G.A.Briscoe, E.L.Norton, S.J.Geier, I.Hung, Shaohui Zheng, J.Autschbach, R.W.Schurko, *J.Am.Chem.Soc.* (2009), **131**, 3317.
COACAC03	tris(acetylacetonato) cobalt(III)	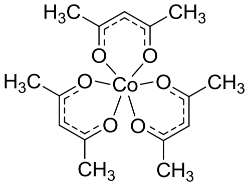	G.J.Kruger, E.C.Reynhardt, *Acta Crystallogr.,Sect.B:Struct.Crystallogr.Cryst.Chem.* (1974), **30**, 822.
COSRUX	(o-catecholato-O,O')-(η^5-pentamethyl-cyclopentadienyl)-cobalt(III)	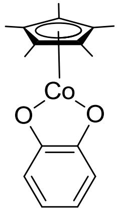	E.J.Miller, A.L.Rheingold, T.B.Brill, *J.Organomet.Chem.* (1984), **273**, 377.

CPCHTI	(η^5-cyclopentadienyl)-(η^7-cycloheptatrienyl)-titanium	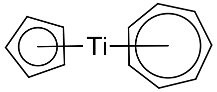	J.D.Zeinstra, J.L.de Boer, *J.Organomet.Chem.* (1973), **54**, 207.
CPMNCO01	tricarbonyl-(η^5-cyclopentadienyl)-manganese(I)	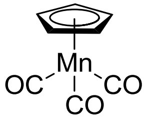	P.J.Fitzpatrick, Y.Le Page, J.Sedman, I.S.Butler, *Inorg.Chem.* (1981), **20**, 2852.
CPTIBP01	(2,2'-bipyridyl)-bis(η^5-cyclopentadienyl)-titanium	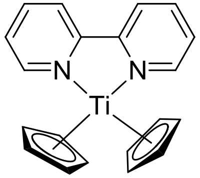	R.Gyepes, P.T.Witte, M.Horacek, I.Cisarova, K.Mach, *J.Organomet.Chem.* (1998), **551**, 207.
DADHIZ	dicarbonyl-chloro-(η^5-cyclopentadienyl)-iron(II)	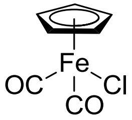	K.-J.Jens, E.Weiss, *Acta Crystallogr.,Sect.C:Cryst.Struct.Commun.* (1985), **41**, 895.
DBENCR10	bis(η^6-benzene)-chromium(0)	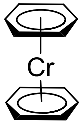	F.Jellinek, *J.Organomet.Chem.* (1963), **1**, 43.
DCPVCL	chloro-dicyclopentadienyl-vanadium	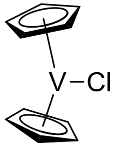	B.F.Fieselmann, G.D.Stucky, *J.Organomet.Chem.* (1977), **137**, 43.

DIGWUL	chloro-(2,9-dimethyl-1,10-phenanthroline-N,N')-copper(I)	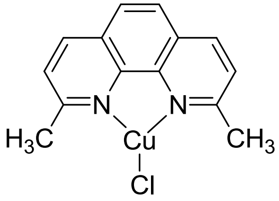	P.C.Healy, C.Pakawatchai, A.H.White, *J.Chem.Soc.,Dalton Trans.* (1985), 2531.
FEROCE06	ferrocene	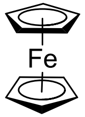	F.Takusagawa, T.F.Koetzle, *Acta Crystallogr.,Sect.B:Struct.Crystallogr.Cryst.Chem.* (1979), **35**, 1074.
FEROCE27	ferrocene	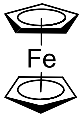	C.P.Brock, Yigang Fu, *Acta Crystallogr.,Sect.B:Struct.Sci.* (1997), **53**, 928.
FOHCOU	hexacarbonyl-chromium(0)	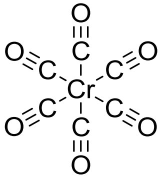	A.Whitaker, J.W.Jeffery, *Acta Crystallogr.* (1967), **23**, 977.
FOMKIB01	bis(N-isopropylsalicylaldiminato-N,O)-zinc(II)	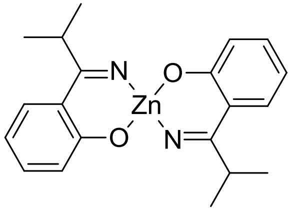	F.A.Bottino, P.Finocchiaro, E.Libertini, G.Mattern, *Z.Kristallogr.* (1989), **187**, 71.

FURRAL	dicarbonyl-(η^5-cyclopentadienyl)-(2,2'-bipyridine-N,N')-vanadium	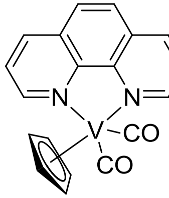	S.Gambarotta, A.Chiesi-Villa, C.Guastini, *Inorg.Chem.* (1988), **27**, 99.
GIHWIE01	chloro-(tris(2-pyridylmethyl)amine)-copper(I)	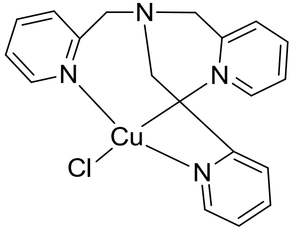	S.C.N.Hsu, S.S.C.Chien, H.H.Z.Chen, M.Y.Chiang, *J.Chin.Chem.Soc.(Taipei)* (2007), **54**, 685.
HEPSOL	(1,3-di-t-butyl-2,3-dihydroimidazol-2-ylidene)-(η^5-cyclopentadienyl)-copper(I)	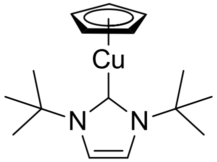	Hongping Ren, Xi Zhao, Shansheng Xu, Haibin Song, Baiquan Wang, *J.Organomet.Chem.* (2006), **691**, 4109.
JAFNAG	tetracarbonyl-(η^5-pentamethyl-cyclopentadienyl)-vanadium	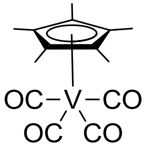	M.Herberhold, A.-M.Dietel, W.Milius, *Z.Naturforsch.,B:Chem.Sci.* (2003), **58**, 299.
JARNAS	(acetylacetonato)-dichloro-(η^5-cyclopentadienyl)-titanium(IV)	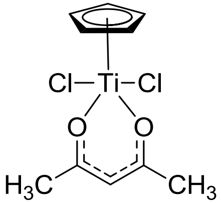	Samkeun Lee, Kyoung-Chae Seo, Sock Sung Yun, Sung Kwon Kang, *J.Coord.Chem.* (2005), **58**, 695.

NCKLCN	nickelocene	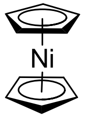	P.Seiler, J.D.Dunitz, *Acta Crystallogr.,Sect.B:Struct.Crystallogr.Cryst.Chem.* (1980), **36**, 2255.
NEZWEU	(1,4,7-trimethyl-1,4,7-triazacyclononane)-trichloro-scandium	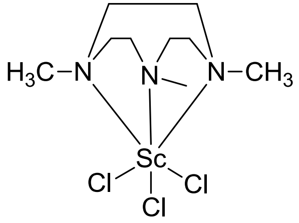	S.Hajela, W.P.Schaefer, J.E.Bercaw, *J.Organomet.Chem.* (1997), **532**, 45.
NIDCAR06	bis(N,N-diethyldithiocarbamato-S,S')-nickel(II)	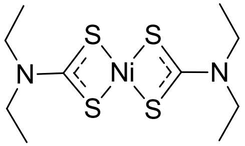	A.Husain, S.A.A.Nami, S.P.Singh, M.Oves, K.S.Siddiqi, *Polyhedron* (2011), **30**, 33.
NISALO01	bis(salicylaldoximato-N,O)-nickel(II)	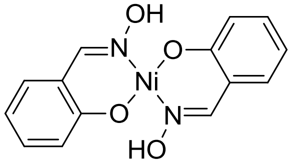	K.Mereiter, *CSD Communication(Private Communication)*
PESKEE01	bis(benzoato-O,O')-(2,2'-bipyridine-N,N')-zinc(ii)	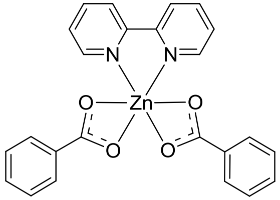	Geun Hee Eom, Hyun Min Park, Min Young Hyun, Seung Pyo Jang, Cheal Kim, Jun Ho Lee, Suk Joong Lee, Sung-Jin Kim, Youngmee Kim, *Polyhedron* (2011), **30**, 1555.

PMCCOC	dicarbonyl-(pentamethyl-cyclopentadienyl)-cobalt(I)	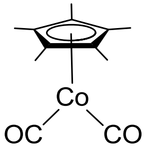	L.R.Byers, L.F.Dahl, *Inorg.Chem.* (1980), **19**, 277.
PVVBAY01	bis(acetylacetonato)-diaqua-Iron(II)	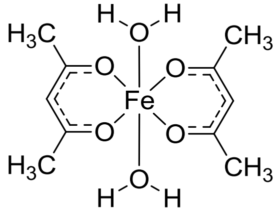	J.Laugier, J.P.Mathieu, *Acta Crystallogr.,Sect.B:Struct.Crystallogr.Cryst.Chem.* (1975), **31**, 631.
QENJAV	cis-dichloro-(η^5-pentamethylcyclopentadienyl)-(2,4-pentanedionato)-titanium(IV)	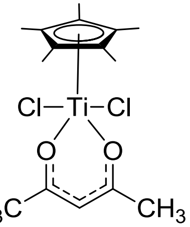	Samkeun Lee, Sung Kwon Kang, Seung Jae Lee, Sock Sung Yun, Tae-Sun Chang, *Bull.Korean Chem.Soc.* (2005), **26**, 1620.
RUJBUT	carbonyl-chloro-(2,2'-dipyridylamine)-copper(I)	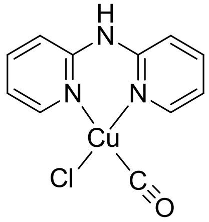	F.Ugozzoli, A.M.M.Lanfredi, N.Marsich, A.Camus, *Inorg.Chim.Acta* (1997), **256**, 1.

SAYVOE	bis(Nitrito-N)-(1,10-phenanthroline-N,N')-(2,4-pentanedionato-O,O')-cobalt(III)	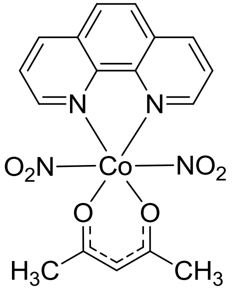	Yun-Long Feng, Hong Lin, *Z.Kristallogr.-New Cryst.Struct.* (2005), **220**, 199.
TCBMNI	bis(N,N-dimethyldithiocarbamato)-nickel(II)	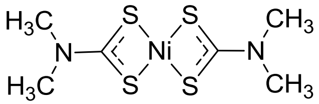	J.Lokaj, J.Garaj, V.Kettmann, V.Vrabel, *Collect.Czech.Chem.Commun.* (1980), **45**, 2147.
TESBUO	(η^5-cyclopentadienyl)-tris(acetato-O,O')-titanium	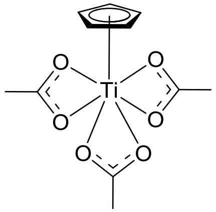	Yujin Huang, N.Etkin, R.R.Heyn, T.T.Nadasdi, D.W.Stephan, *Organometallics* (1996), **15**, 2320.
TITODC10	dicarbonyl-dicyclopentadienyl-titanium(II)	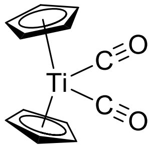	J.L.Atwood, K.E.Stone, H.G.Alt, D.C.Hrncir, M.D.Rausch, *J.Organomet.Chem.* (1977), **132**, 367.

TROPSC	tris(tropolonato) scandium(III)	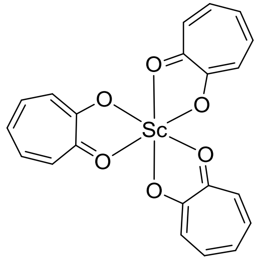	T.J.Anderson, M.A.Neuman, G.A.Melson, *Inorg.Chem.* (1974), **13**, 158.
VANPAB	bis(η⁵-pentamethyl-cyclopentadienyl)-(toluato-O,O')-scandium	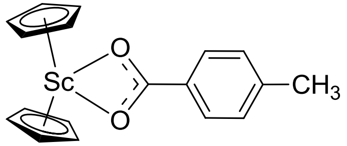	M.A.St.Clair, B.D.Santarsiero, *Acta Crystallogr.,Sect.C:Cryst.Struct.Commun.* (1989), **45**, 850
VIPGEG	tricarbonyl-(η⁵-pentamethyl-cyclopentadienyl)-manganese(I)	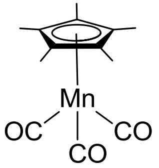	S.Fortier, M.C.Baird, K.F.Preston, J.R.Morton, T.Ziegler, T.J.Jaeger, W.C.Watkins, J.H.MacNeil, K.A.Watson, K.Hensel, Y.Le Page, J.-P.Charland, A.J.Williams, *J.Am.Chem.Soc.* (1991), **113**, 542
YABGAK	dichloro-(6,6'-dimethyl-2,2'-bipyridyl)-iron(II)	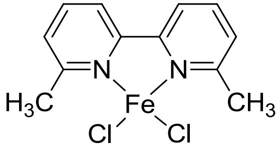	B.C.K.Chan, M.C.Baird, *Inorg.Chim.Acta* (2004), **357**, 2776,
ZOSWEJ	chloro-pentacarbonyl-manganese(I)	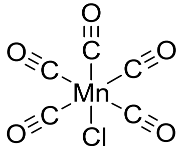	P.T.Greene, R.F.Bryan, *J.Chem.Soc.A* (1971), 1559,

AJUJIY03	tris(8-quinolinolato-N,O)-cobalt(III) methanol solvate	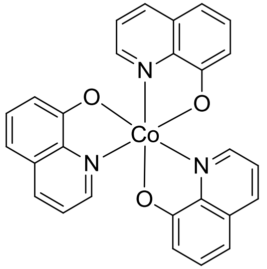	D.Mandal, M.Mikuriya, Hoong-Kun Fun, D.Ray, *Inorg.Chem.Commun.* (2007), **10**, 657.
BOVNUW	(6,6'-dimethoxy-2,2'-[cyclohexane-1,2-diylbis(nitrilomethylidyne)]diphenolato-κ[4]O[1],N,N',O[1'])-iron(II) monohydrate	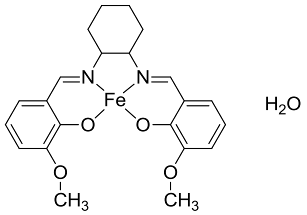	Peng-Fei Yan, Yan Bao, Hong-Feng Li, Guang-Ming Li, *Acta Crystallogr.,Sect.E:Struct.Rep.Online* (2009), **65**, m832.
CAPVUM	(2-(((2-((4-(hydroxy)pent-3-en-2-ylidene)amino)ethyl)imino)methyl)phenolato)-nickel methanol solvate	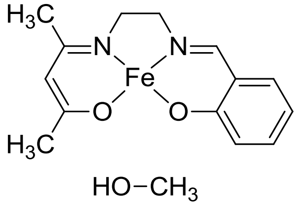	Jia-Zhong Wang, Jing-Chi Gu, Xue-Cheng Zhang, Jing Huang, Bi-Xue Zhu, *Wuji Huaxue Xuebao(Chin.)(Chin.J.Inorg.Chem.)* (2012), **28**, 321.
CECGIB	(η^6-1,4-dihydroxynaphthalene)-tricarbonyl-chromium benzene solvate	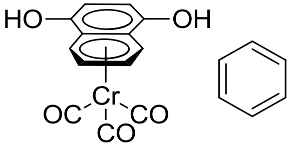	E.P.Kundig, A.E.Garcia, T.Lomberget, G.Bernardinelli, *Angew.Chem.,Int.Ed.* (2006), **45**, 98.

CEKHOP	2,2'-bipyridine-chloro-triphenylphosphine-copper(i) monohydrate	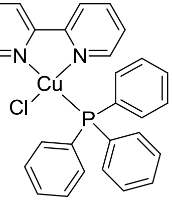	B.E.Green, C.H.L.Kennard, G.Smith, B.D.James, A.H.White, *Acta Crystallogr.,Sect.C:Cryst.Struct.Commun.* (1984), **40**, 426.
COXSOZ	chloro-(1,10-phenanthroline)-(triphenylphosphine)-copper 1,2-dichloroethane solvate	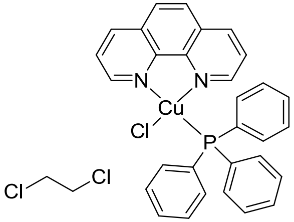	L.I.Panferova, F.M.Miloserdov, Anton Lishchynskyi, M.M.Belmonte, J.Benet-Buchholz, V.V.Grushin, *Angew.Chem.,Int.Ed.* (2015), **54**, 5218.
DARCIK	(benzene-1,2-diolato)-(2,2'-(1,2-phenylenebis((nitrilo)methylylidene))bis(4-methylphenolato))-titanium dichloromethane solvate	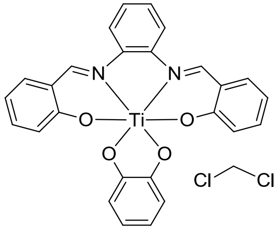	A.Tzubery, E.Y.Tshuva, *Inorg.Chem.* (2012), **51**, 1796.
DOQGOH	bis(4,4,4-trifluoro-3-((2-methylquinolin-8-yl)amino)-1-phenylbut-2-en-1-onato)-zinc diethyl ether solvate	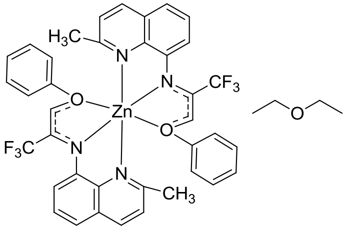	K.A.Gerling, N.M.Rezayee, A.L.Rheingold, D.B.Green, J.M.Fritsch, *Dalton Trans.* (2014), **43**, 16498.

FIQXAG	bromo-mesityl-(1,10-phenanthroline)-nickel tetrahydrofuran solvate	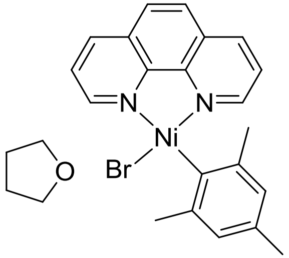	D.G.Yakhvarov, A.Petr, V.Kataev, B.Buchner, S.Gomez-Ruiz, E.Hey-Hawkins, S.V.Kvashennikova, Y.S.Ganushevich, V.I.Morozov, O.G.Sinyashin, *J.Organomet.Chem.* (2014), **750**, 59.
FIXJON	1,1'-diisocyanatoferrocene benzene solvate	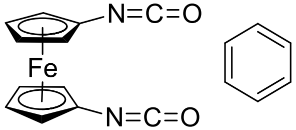	A.R.Petrov, K.Jess, M.Freytag, P.G.Jones, M.Tamm, *Organometallics* (2013), **32**, 5946.
GAPMIT	tris(2-(o-hydroxyphenyl)-pyridine-O,N)-cobalt(III) dichloromethane solvate	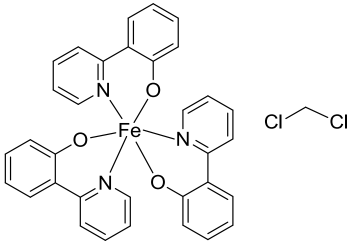	P.Ganis, A.Saporito, A.Vitagliano, G.Valle, *Inorg.Chim.Acta* (1988), **142**, 75.
JEVMIH	bis(2-(4,5-dihydro-1H-imidazol-2-yl)phenolato-N,O)-zinc(ii) methanol solvate	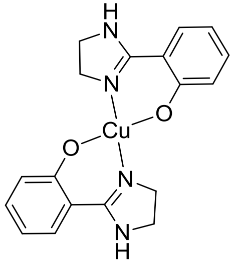	Hong-Shan He, *Acta Crystallogr.,Sect.E:Struct.Rep.Online* (2007), **63**, m344.

KUHRIQ	(acetylacetonato)-(2,2'-(ethane-1,2-diylbis(nitrilomethylylidene))bis(6-methoxyphenolato))-cobalt(III) dimethyl sulfoxide solvate	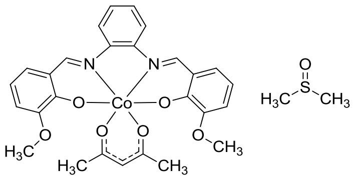	C.Schieber, J.Howitt, U.Putz, J.M.White, C.L.Parish, P.S.Donnelly, Seong-Seng Tan, *J.Biol.Chem.* (2011), **286**, 8555.
MAHWAV	tricarbonyl-(η^6-1,3-diphenylurea)-chromium ethanol solvate	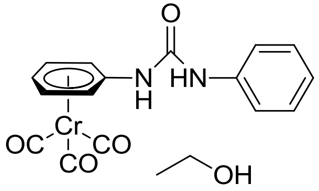	Xiaohui Tian, Li Song, Yizhong Yuan, Jiaping Lin, *Organometallics* (2010), **29**, 509.
MOLPOT	(η^5-(4-t-butyl-1-methyl-1-cyclohexyl)cyclopentadienyl)-dichloro-(η^5-cyclopentadienyl)-titanium(IV) dichloromethane solvate	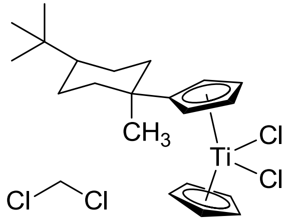	A.Gansauer, I.Winkler, D.Worgull, D.Franke, T.Lauterbach, A.Okkel, M.Nieger, *Organometallics* (2008), **27**, 5699.
OVIMAI	tris(2-aminoquinolin-8-olato)-scandium 1,2-dimethoxyethane solvate	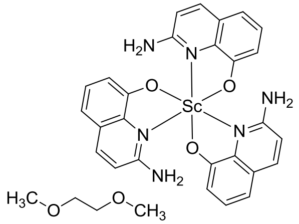	M.A.Katkova, T.V.Balashova, A.P.Pushkarev, I.Yu.Ilyin, G.K.Fukin, E.V.Baranov, S.Yu.Ketkov, M.N.Bochkarev, *Dalton Trans.* (2011), **40**, 771.

PEKNOK	(2-(((2,6-dimethylphenyl)imino)methyl)-N,N-dimethylaniline)-dibromo-nickel dichloromethane solvate	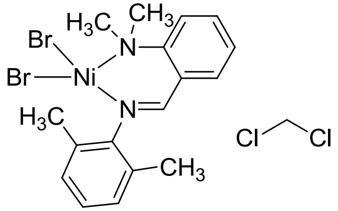	Jun-Kai Zhang, Peng-Chao Wang, Xiao-Wei Wang, Liang Wang, Jun-Cai Chen, Zhi-Wu Zheng, Yong-Ping Niu, *J.Organomet.Chem.* (2011), **696**, 3697.
QOFHOI	tris(8-oxyquinoline)-vanadium(III) methanol solvate	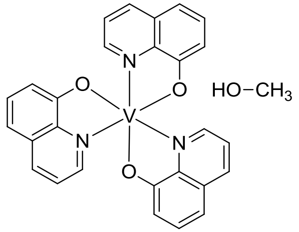	M.J.Manos, A.J.Tasiopoulos, C.Raptopoulou, A.Terzis, J.D.Woollins, A.M.Z.Slawin, A.D.Keramidas, T.A.Kabanos, *J.Chem.Soc.,Dalton Trans.* (2001), 1556.

Table S5 Crystal structure details for 27 open shell pairs.

ACACCR07	tris(acetylacetonato)-chromium(III)	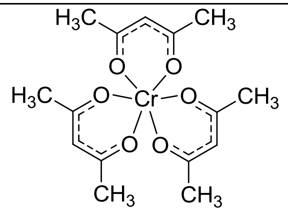	L.S.von Chrzanowski, M.Lutz, A.L.Spek, *Acta Crystallogr.,Sect.C:Cryst.Struct.Commun.* (2007), **63**, m377.
ACACCU02	bis(acetylacetonato)-copper(II)	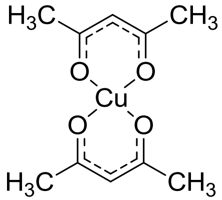	G.Berry, G.Callon, B.Gowans, J.N.Low, R.Smith, *CSD Communication(Private Communication)* (2004)
ACACVO04	bis(acetylacetonato)-oxo-vanadium(IV)	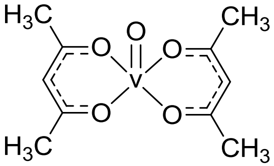	M.Hoshino, A.Sekine, H.Uekusa, Y.Ohashi, *Chem.Lett.* (2005), **34**, 1228.
AFALID01	ferrocenium tetrafluoroborate	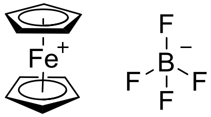	S.R.Posner, L.C.Lorson, A.R.Gell, B.M.Foxman, *Cryst.Growth Des.* (2015), **15**, 3407.
ACACMN2 1	tris(acetylacetonato)-manganese(III)	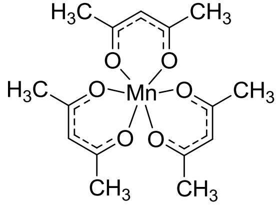	B.R.Stults, R.S.Marianelli, V.W.Day, *Inorg.Chem.* (1979), **18**, 1853.

AFATAE	chloro-(2,2'-(1,2-phenylenebis((nitrilo)methylylidene))diphenolato)-manganese(III) methanol solvate	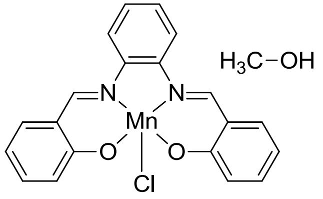	H.Lin, J.-G.Wang, H.-T.Shi, Q.Chen, Q.-F.Zhang, *Acta Crystallogr.,Sect.E:Struct.Rep.Online* (2013), **69**, m404.
AGEFEX	ferrocenium tris(hexafluoroacetylacetonato)-manganese(II)	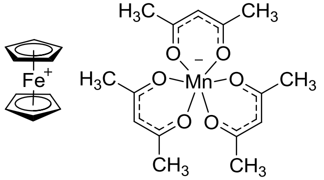	J.R.Bryant, J.E.Taves, J.M.Mayer, *Inorg.Chem.* (2002), **41**, 2769.
BEPRET	bis(dibenzoylmethane)-chloro-manganese(III) dichloromethane solvate	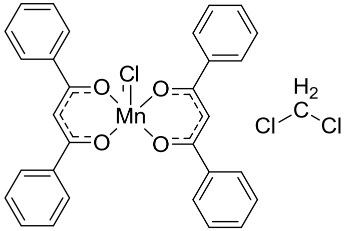	G.Aromi, M.J.Knapp, J.-P.Claude, J.C.Huffman, D.N.Hendrickson, G.Christou, *J.Am.Chem.Soc.* (1999), **121**, 5489.
CIRSEB	cyano-bis(η^5-pentamethyl-cyclopentadienyl)-vanadium	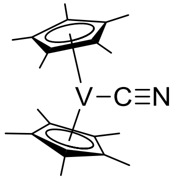	S.Gambarotta, C.Floriani, A.Chiesi-Villa, C.Guastini, *Inorg.Chem.* (1984), **23**, 1739.
CPNDYV07	bis(η^5-cyclopentadienyl)-vanadium	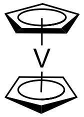	A.L.Spek, *CSD Communication(Private Communication)* (2000)

CUBEAC01	bis(benzoylacetonato)-copper(II)	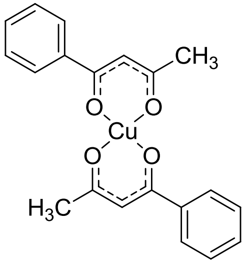	S.K.Dey, B.Bag, Zhongyuan Zhou, A.S.C.Chan, S.Mitra, *Inorg.Chim.Acta* (2004), **357**, 1991.
CUQUIN05	bis(8-hydroxyquinolinato-N,O)-copper(II)	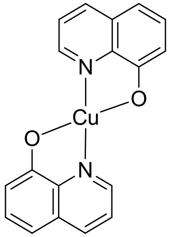	S.Ammor, G.Coquerel, G.Perez, F.Robert, *Eur.J.Solid State Inorg.Chem.* (1992), **29**, 445.
DERNOD05	bis(2,2,6,6-tetramethyl-3,5-heptanedionato)-copper(II)	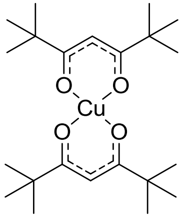	M.Hamid, M.Mazhar, M.Zeller, A.D.Hunter, *CSD Communication(Private Communication)* (2005)
DMTCCU	bis(N,N-dimethyldithiocarbamate)-copper(II)	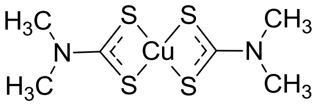	F.W.B.Einstein, J.S.Field, *Acta Crystallogr.,Sect.B:Struct.Crystallogr.Cryst.Chem.* (1974), **30**, 2928.

FEACAC03	tris(acetylacetonato)-iron(III)	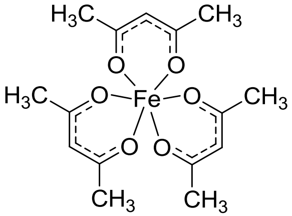	M.-L.Hu, Z.-M.Jin, Q.Miao, L.-P.Fang, *Z.Kristallogr.-New Cryst.Struct.* (2001), **216**, 597.
HIDPOZ	(acetylacetonato-O,O')-titanocene(III)	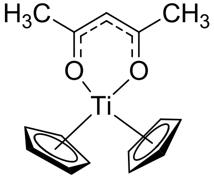	A.M.Bond, R.Colton, U.Englert, H.Hugel, F.Marten, *Inorg.Chim.Acta* (1995), **235**, 117.
HIRNEB	4-(4-fluorophenyl)-methyleneamino-2,2,6,6-tetramethylpiperidin-1-oxyl radical	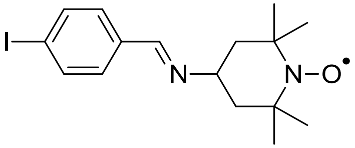	F.Iwasaki, J.H.Yoshikawa, H.Yamamoto, E.Kan-nari, K.Takada, M.Yasui, T.Ishida, T.Nogami, *Acta Crystallogr.,Sect.B:Struct.Sci.* (1999), **55**, 231.
IGAGEC	tris(1,1,1,5,5,5-hexafluoroacetylacetonato)-chromium(III)	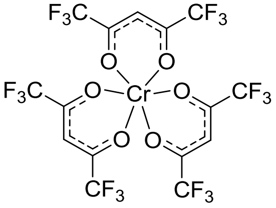	P.G.Jessop, M.M.Olmstead, C.D.Ablan, M.Grabenauer, D.Sheppard, C.A.Eckert, C.L.Liotta, *Inorg.Chem.* (2002), **41**, 3463.
IPEZOS	bis(2,2,6-trimethylheptane-3,5-dionato)-copper(II)	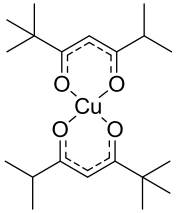	M.J.Delarosa, K.S.Bousman, J.T.Welch, P.J.Toscano, *J.Coord.Chem.* (2003), **56**, 1339.

JIYKEH01	perchlorotrityl radical	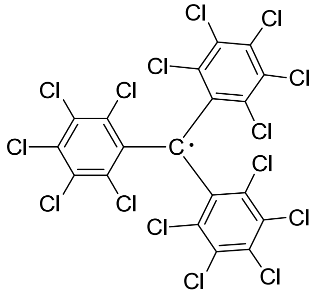	J.Guasch, X.Fontrodona, I.Ratera, C.Rovira, J.Veciana, *Acta Crystallogr., Sect.C:Cryst.Struct.Commun.* (2013), **69**, 255.
MACACU10	bis(3-methylpentane-2,4-dionato)-copper(II)	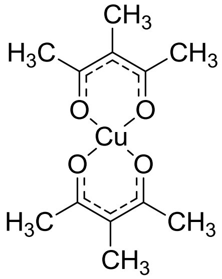	I.Robertson, M.R.Truter, *J.Chem.Soc.A* (1967), 309.
POXYUW	rac-trans-2,6-dimethyl-2,6-diphenylpiperidine N-oxide	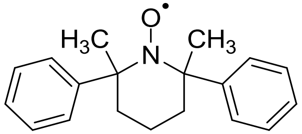	J.Einhorn, C.Einhorn, F.Ratajczak, A.Durif, M.-T.Averbuch, J.-L.Pierre, *Tetrahedron Lett.* (1998), **39**, 2565.
QQQBWP03	trans-bis(1,1,1-trifluoropentan-2,4-dionato)-copper(ii)	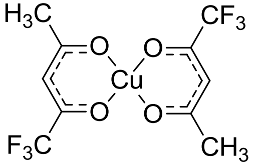	R.E.Marsh, *Acta Crystallogr.,Sect.B:Struct.Sci.* (2009), **65**, 782.

SUZSAH	(acetylacetonato-O,O')-bis(picolinato-N,O)-manganese(III)	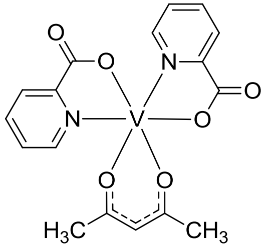	T.Nakamura, K.Niwa, S.Usugi, H.Asada, M.Fujiwara, T.Matsushita, *Polyhedron* (2001), **20**, 191
TMPPIO13	2,2,6,6-tetramethylpiperidine-1-oxyl	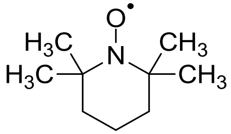	S.Spirk, F.Belaj, T.Madl, R.Pietschnig, *Eur.J.Inorg.Chem.* (2010), 289.
VAACAC02	tris(2,4-pentanedionato-O,O')-vanadium(III)	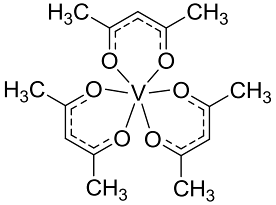	C.A.L.Filgueiras, A.Horn Junior, R.A.Howie, J.M.S.Skakle, J.L.Wardell, *Acta Crystallogr.,Sect.E:Struct.Rep.Online* (2001), **57**, m157
YUJYIL01	4-((4-phenylbenzylidene)amino)-2,2,6,6-tetramethylpiperidine-1-oxyl radical	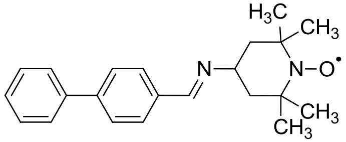	F.Iwasaki, J.H.Yoshikawa, H.Yamamoto, E.Kan-nari, K.Takada, M.Yasui, T.Ishida, T.Nogami, *Acta Crystallogr.,Sect.B:Struct.Sci.* (1999), **55**, 231
